# A combined physical–chemical and microbiological approach to unveil the fabrication, provenance, and state of conservation of the *Kinkarakawa-gami* art

**DOI:** 10.1038/s41598-020-73226-6

**Published:** 2020-10-02

**Authors:** Elena Piacenza, Alessandro Presentato, Francesca Di Salvo, Rosa Alduina, Vittorio Ferrara, Valeria Minore, Antonio Giannusa, Giuseppe Sancataldo, Delia Francesca Chillura Martino

**Affiliations:** 1 National Interuniversity Consortium of Materials Science and Technology (INSTM), UdR of Palermo, 50121 Florence, Italy; 2 Department of Biological, Chemical and Pharmaceutical Sciences and Technologies (STEBICEF), University of Palermo, 90128 Palermo, Italy; 3 Department of Agricultural, Food and Forest Sciences, University of Palermo, 90128 Palermo, Italy; 4 Vincenzo Ragusa and O’Tama Kiyohara Museum, Liceo Artistico Statale Vincenzo Ragusa e O’Tama Kiyohara, 90129 Palermo, Italy; 5 Department of Physics and Chemistry (DIFC) Emilio Segrè, University of Palermo, 90128 Palermo, Italy

**Keywords:** Microbiology, Materials science

## Abstract

*Kinkarakawa-gami* wallpapers are unique works of art produced in Japan between 1870 and 1905 and exported in European countries, although only few examples are nowadays present in Europe. So far, neither the wallpapers nor the composing materials have been characterised, limiting the effective conservation–restoration of these artefacts accounting also for the potential deteriogen effects of microorganisms populating them. In the present study, four *Kinkarakawa-gami* wallpapers were analysed combining physical–chemical and microbiological approaches to obtain information regarding the artefacts’ manufacture, composition, dating, and their microbial community. The validity of these methodologies was verified through a fine *in blind* statistical analysis, which allowed to identify trends and similarities within these important artefacts. The evidence gathered indicated that these wallpapers were generated between 1885 and 1889, during the so-called industrial production period. A wide range of organic (proteinaceous binders, natural waxes, pigments, and vegetable lacquers) and inorganic (tin foil and pigments) substances were used for the artefacts’ manufacture, contributing to their overall complexity, which also reflects on the identification of a heterogeneous microbiota, often found in Eastern environmental matrices. Nevertheless, whether microorganisms inhabiting these wallpapers determined a detrimental or protective effect is not fully elucidated yet, thus constituting an aspect worth to be explored to deepen the knowledge needed for the conservation of *Kinkarakawa-gami* over time.

## Introduction

*Kinkarakawa-gami* represents an important example of Japanese art and its mixture within the European culture between the XIX and XX centuries. This manufacture derived from the importation in Japan of the so-called *Kinkarakawa* (i.e., foreign gilt leather) by the Dutch East India Company, which was one of the few foreign companies allowed to commercially trade with this eastern country up to 1854^1^. Subsequently, the opening of Japan to the western world encouraged the exportation of authentic original Japanese goods, among which *Kinkarakawa-gami* (i.e., gilt leather-like wallpapers) gained international relevance and recognition, mostly with British, Dutch and German companies^1,2^.

Several European sources of the XIX century documented the traditional production stream of *Kinkarakawa-gami*, identifying four steps: (1) hand-making of the leather-like paper, (2) its embossing with a chosen pattern, (3) gilding of the paper with metal powders or foils, and (4) its final decoration^3–6^. Historically, three periods of *Kinkarakawa-gami*’s manufacture are distinguished: (1) the Japonist Enlightenment (1873–1884), during which the wallpapers were entirely hand-made, properly depicting Japanese patterns and featuring an “oily smell”^7^, which did not encounter the European consumer taste; (2) the period of industrial production (1882–1889), when several British factories were established on Japanese soil, leading to faster yet traditional manufacture, as well as a change in the wallpaper design; and (3) the mass-produced commodity period (1890–1905), where the patterns were directly chosen by British designers and cheap industrial processes were applied to answer to the large demand of European customers^1^, however resulting in the end—or almost all—of the *Kinkarakawa-gami* art^2^.

The uniqueness of these Japanese wallpapers relies on their limited presence in Europe, therefore physical–chemical studies aimed to unveil the features of these artefacts are still missing, being of paramount importance to develop successful and sound conservation-restoration strategies. This aspect significantly gains importance since, to the best of the authors’ knowledge, *Kinkarakawa-gami* belonging to the *Vincenzo Ragusa and O’Tama Kiyohara* Collection of Palermo (patrimony of the *Liceo Artistico* founded in the XIX century by the sculptor Vincenzo Ragusa) represents the first finding of this form of Japanese fine art in Italy. Thus, four wallpapers of this collection were studied through a multidisciplinary approach aimed to overview their state of conservation for their future restoration, therefore enhancing the artistic value of these artefacts. The wallpaper manufacture was evaluated through a standard operating procedure involving non-invasive and non-destructive physical–chemical techniques—that allow to perform an extensive sampling of the artefacts without altering them or their conservation state^8^—and microscopy observations, focusing on the (1) paper composition, (2) metallic layer on the recto of these artefacts, identification of (3) inorganic pigments, (4) organic pigments, and (5) substances (e.g., lacquers) exploited as binders for the pigments^9^. Physical–chemical results were validated through *in blind* statistical analyses, which, by identifying homogeneous clusters of curves with respect to their shape, further unveiled important differences and similarities among the four artefacts. Finally, the cultivable microbiota populating these wallpapers and their potential interaction with both inorganic and organic substrates were critically discussed to better understand how to properly restore and preserve these *Kinkarakawa-gami* through time.

## Results and discussion

### Paper composition

**Figure 1:**
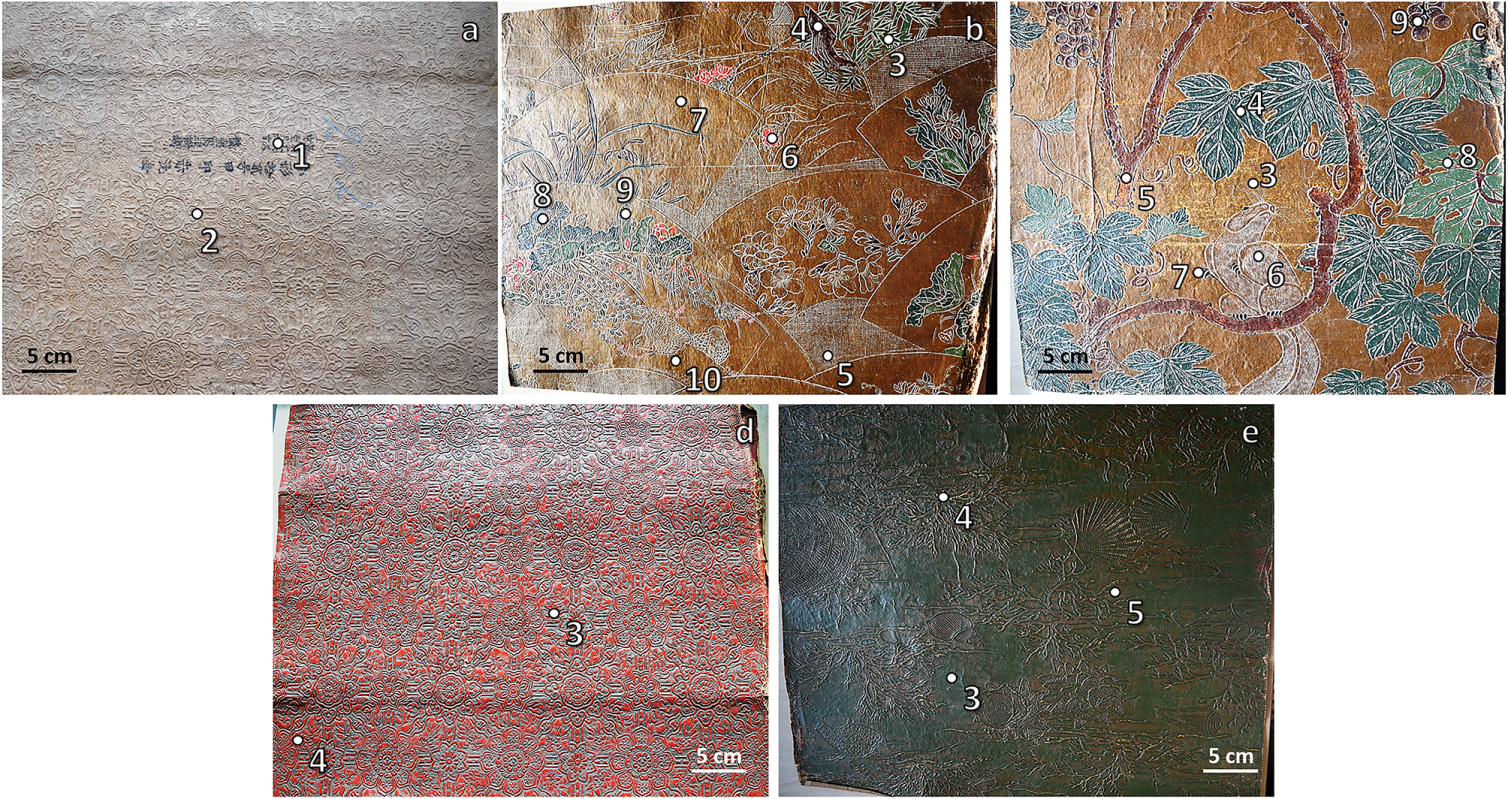
*Kinkarakawa-gami* wallpapers. In (**a**) is depicted the verso of one of the wallpapers and the points 1 (written portion) and 2 (non-written portion) that were chosen—for all the artefacts—for the analyses, while the wallpapers’ recto and the investigated sampling points (numbers) are represented in (**b**) INV_11 (3–10), (**c**) INV_13 (3–9), (**d**) INV_15 (3–4), and (**e**) INV_20 (3–5).

**Figure 2:**
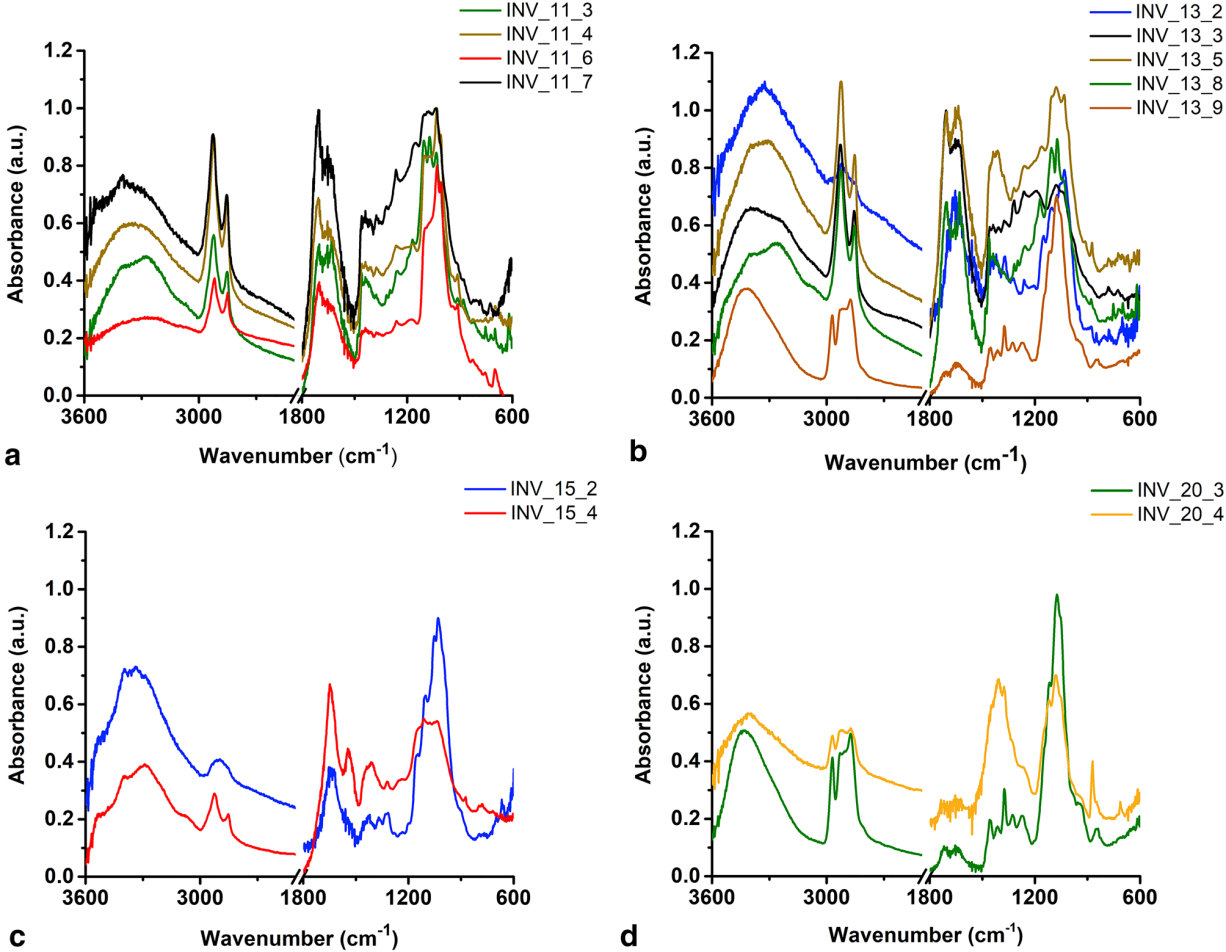
µATR-FTIR spectra performed on the wallpapers. The spectra were collected on the selected sampling points (as indicated in the legend) of (**a**) INV_11, (**b**) INV_13, (**c**) INV_15, and (**d**) INV_20. For clarity, the µATR-FTIR spectra were offset of 0.10 arbitrary units (a.u.).

**Figure 3:**
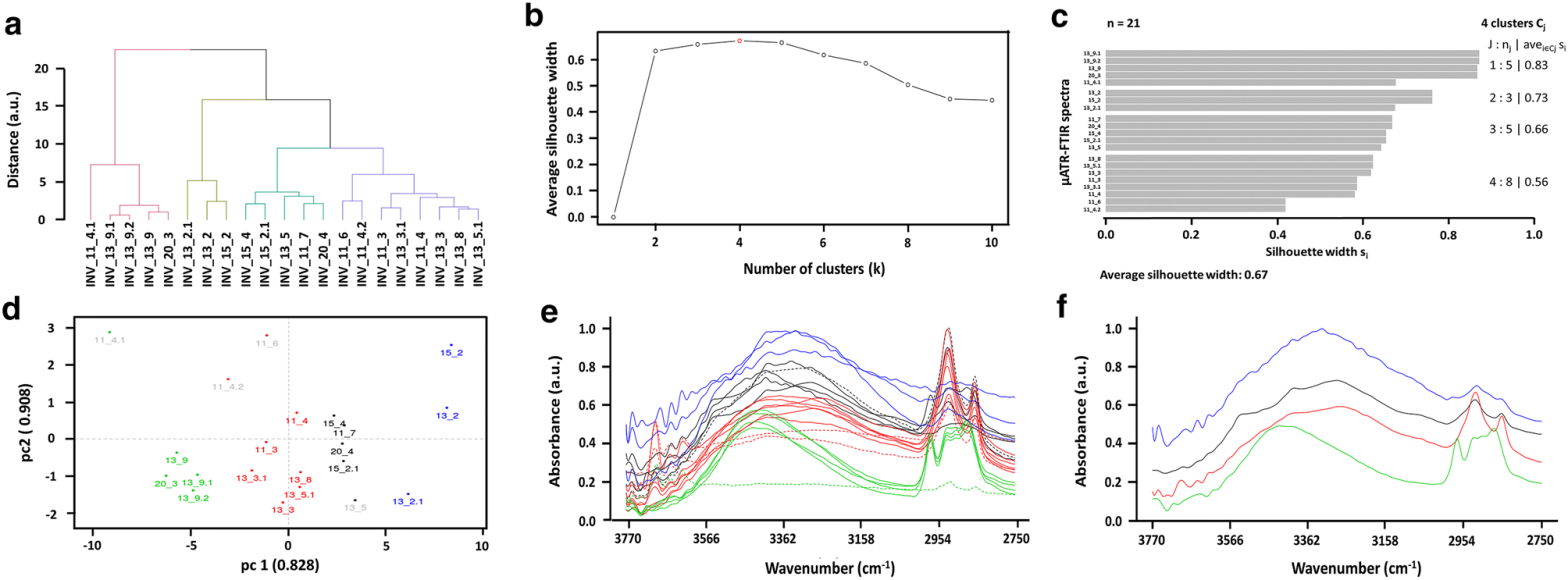
Statistical analysis performed on µATR-FTIR spectra for the 3770–2750 cm^−1^ interval. The hierarchical relationship between the spectra is represented by the dendrogram depicted in (**a**), while (**b**, **c**) show the estimation of the number of clusters using the average silhouette width and the obtained silhouette of clusters, respectively. PCA results, µATR-FTIR spectra, and the most representative (i.e., deepest) curve for each identified cluster (highlighted with different colours) are illustrated in (**d**–**f**). For clarity, the most external spectra of the clusters are underlined in (**d**) with the grey colour.

**Figure 4:**
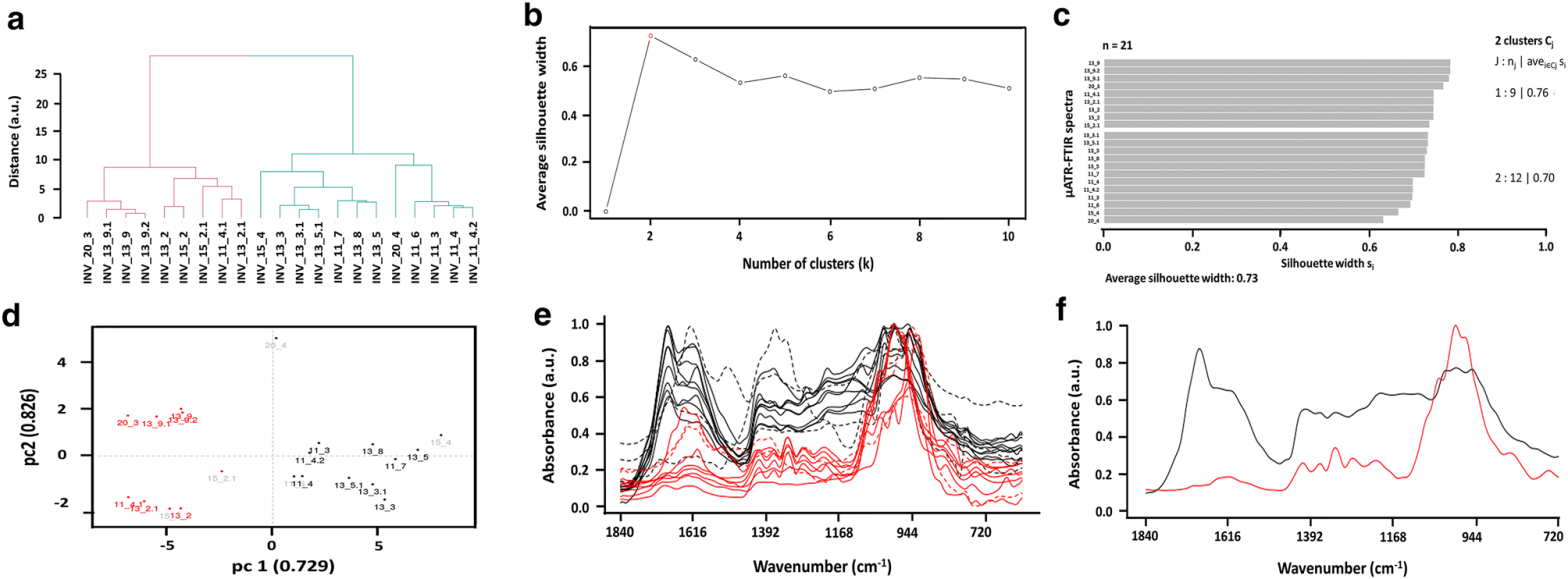
Statistical analysis performed on µATR-FTIR spectra for the 1840–719 cm^−1^ interval. The hierarchical relationship between the spectra is represented by the dendrogram depicted in (**a**), while (**b**, **c**) show the estimation of the number of clusters using the average silhouette width and the obtained silhouette of clusters, respectively. PCA results, µATR-FTIR spectra, and the most representative (i.e., deepest) curve for each identified cluster (highlighted with different colours) are illustrated in (**d**–**f**). For clarity, the most external spectra of the clusters are underlined in (**d**) with the grey colour.

**Figure 5:**
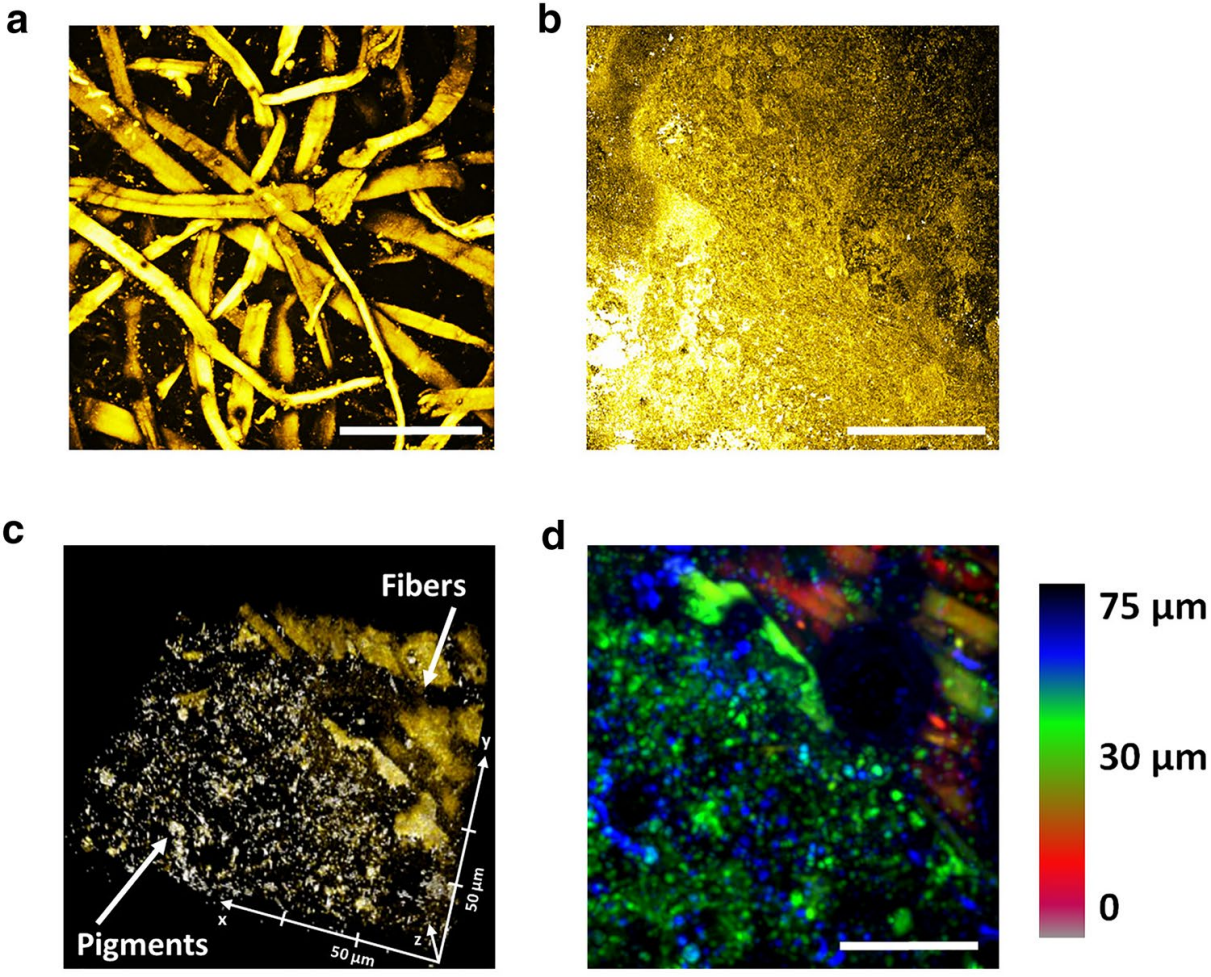
Fluorescence microscopy of *Kinkarakawa-gami* wallpapers. CLSM imaging of representative portions of INV_15 on both verso and recto are illustrated in (**A**, **B**) respectively, while (**C**) depicts a representation of the 3D reconstruction performed on INV_15 recto, whose multilayer structure is quantitatively reported in depth-colour code (analysis depth 75 µm) in (**D**). Scale bars are 100 µm.

**Figure 6:**
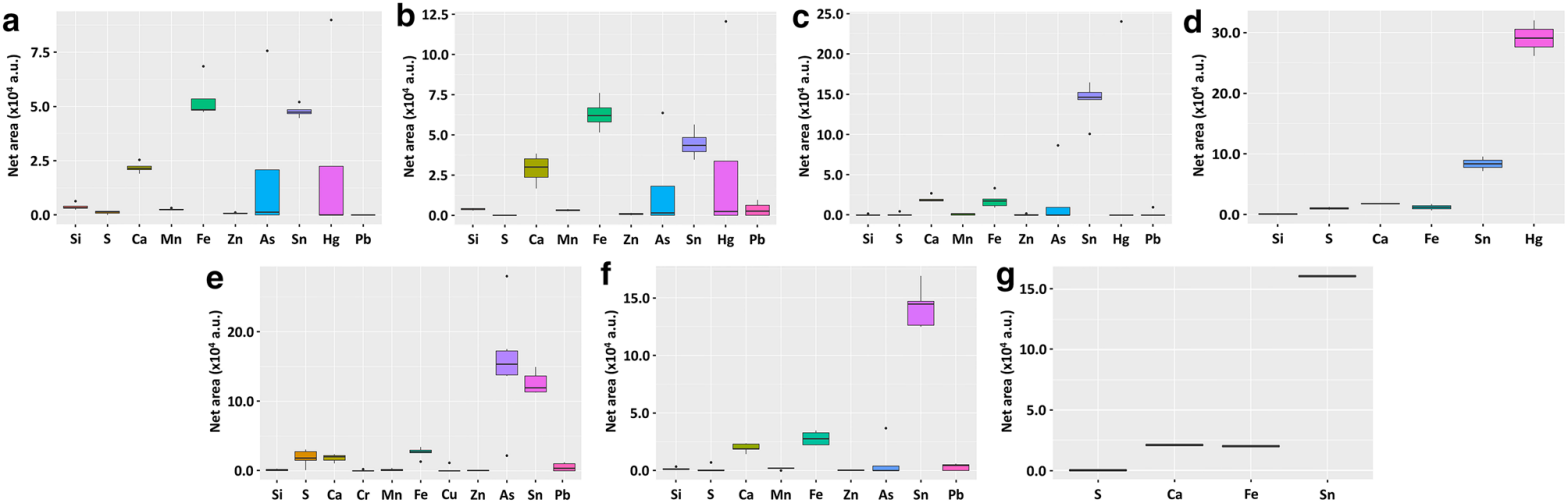
Elemental composition and distribution of *Kinkarakawa-gami* wallpapers obtained through XRF spectroscopy. XRF results are depicted by comparing artefacts’ sampling points collected for (**a**) verso (INV_11_2, INV_13_2, INV_15_2, INV_20_2), (**b**) written portions of verso (INV_11_1, INV_13_1, INV_15_1, INV_20_1) and (**c**) recto’s background (INV_11_5, INV_11_7, INV_13_3, INV_15_3, INV_20_5), as well as (**d**) red (INV_11_6, INV_15_4), (**e**) green (INV_11_3, INV_11_8, INV_11_9, INV_13_4, INV_13_8, INV_20_3), (**f**) brown (INV_11_4, INV_11_5, INV_13_6, INV_13_7, INV_13_9), and (**g**) black (INV_11_10) drawings on the wallpapers’ recto. Elemental compositions of wallpaper sampling points are reported in terms of Net Area (× 104 a.u.), which represents the integral X-ray emission intensity characteristic of each recognized element.

The support material used by Japanese artisans to produce the *Kinkarakawa-gami* wallpapers (henceforth named as INV_11, INV_13, INV_15, and INV_20; Fig. 1, Table 1) derived from either eastern plants or wood pulp, as indicated by the ubiquitous detection, through micro Attenuated Total Reflectance-Fourier Transform Infrared (µATR-FTIR) spectroscopy, of vibrational modes typical of lignin and cellulose (Fig. 2, Table 2, Supplementary Tables 1–4). The chemical similarity with wood pulp was also suggested by a statistical analysis performed on the µATR-FTIR spectra, which was carried out focusing on the 3770–2750 cm^−1^ and 1840–720 cm^−1^ intervals (Supplementary Fig. 1), where the shape of the curves gave information regarding the essential modes of variability among the spectra and their clustering. The cluster analysis showed the hierarchical relationship between the spectra (Figs. 3a, 4a), based on which the optimal number of clusters and the spectra allocation were determined (Figs. 3b, c, 4b, c). Principal modes of variations were derived to identify cluster effects on the shape of the curves, showing that the clusters were correctly visualized in an R^2^ space, as the first two functional Principal Components (PCs) described the 90.8% and 82.6% of the total variability for the first and second interval, respectively (Figs. 3d, 4d). Specifically, cluster 2 (first interval) and 1 (second interval) grouped the spectra collected for wallpapers’ verso, whose high values of silhouette (average s_i_ = 0.73 and 0.76) and distribution in the R^2^ space through PCA assessed their well-identification within the cluster (Fig. 3c–e, 4c–e). The large PC2 value of the first IR interval of INV_15_2 (Fig. 3d) indicated its greater variability than the other verso spectra, likely due to the presence of multiple –OH stretching modes characteristic of cellulose (Fig. 2c; Supplementary Table 3). The Modified Band Depth (MBD) function was used to identify a representative (i.e., deepest) curve for the clusters (Figs. 3f, 4f) describing the shape of the spectra and arrange them by resemblance from the centre (highest depth) outwards (lowest depth) (Supplementary Fig. 2, 3). INV_13_2 was the deepest curve of cluster 2 (first interval) and the other verso spectra featured high depth values, constituting the core (70%) of the cluster (Supplementary Fig. 2b). An analogous situation was observed for the second interval, although the most representative curve was INV_13_9.1 (Fig. 4f), confirming the high degree of similarity of the wallpapers’ verso. Moreover, morphological and structural features of the wallpapers’ verso were studied through Confocal Laser Scanning Microscopy (CLSM) (Fig. 5), revealing a strong fluorescence signal in the visible-light spectrum, and a tangled matrix of organic fibres with a lateral width between 10 and 20 µm for all the samples (Fig. 5a, Supplementary Video 1), which, along with the cellulose and lignin contributions detected within µATR-FTIR spectra (Supplementary Table 1–4), suggested the vegetable origin of the paper. However, it cannot be unequivocally indicated whether the paper was hand-made or made out of mechanical wood pulp^1^, although the detection of silicon (Si) traces by X-Ray Fluorescence (XRF) spectroscopy in the verso sampling points (Fig. 6a, b) could indicate the use of sandstone grinders for the mechanical generation of the pulp^10^. Further, the detection of Si, calcium (Ca) (Fig. 6a, b), and IR absorption bands typical of silicates or carbonates (Supplementary Table 1–4) might infer the use of quartz, calcite, or chalk as fillers^11^.

**Table 1:** Description of the sampling points of the four analysed wallpapers.

Sampling point	Description
INV_11_1	Written portion of verso
INV_11_2	Verso
INV_11_3	Green bamboo plant
INV_11_4	Brown branch
INV_11_5	Background white pattern
INV_11_6	Red flower
INV_11_7	Background
INV_11_8	Dark green leaf
INV_11_9	Light green leaf
INV_11_10	Brown bird paw
INV_13_1	Written portion of verso
INV_13_2	Verso
INV_13_3	Background
INV_13_4	Dark green leaf
INV_13_5	Brown branch
INV_13_6	Squirrel
INV_13_7	Black squirrel paw
INV_13_8	Light green leaf
INV_13_9	Brown grape
INV_15_1	Written portion of verso
INV_15_2	Verso
INV_15_3	Dark background
INV_15_4	Red pattern
INV_20_1	Written portion of verso
INV_20_2	Verso
INV_20_3	Green background
INV_20_4	Gold coral pattern
INV_20_5	Dark background

**Table 2:** Organic and inorganic substances detected through μATR-FTIR performed on INV_11, INV_13, INV_15, and INV_20.

	11_3	11_4	11_6	11_7	13_2	13_3	13_5	13_8	13_9	15_2	15_4	20_3	20_4
**Type of paper**													
Cellulose	✓	✓	✓	✓	✓	✓	✓	✓	✓	✓	✓	✓	✓
Lignin	✓	✓	✓	✓	✓	✓	✓	✓	✓	✓	✓	✓	✓
**Fillers/inorganic pigments**													
Silicates		✓	✓	✓	✓		✓	✓	✓	✓			✓
Carbonates	✓	✓	✓	✓	✓	✓	✓	✓	✓	✓	✓	✓	✓
**Organic binders**													
Unidentified binders	✓	✓	✓	✓	✓	✓	✓	✓					
Proteinaceous binders										✓	✓		
Waxes									✓			✓	✓
Urushi lacquer	✓			✓		✓		✓		✓	✓		✓
**Organic pigments**													
Indigo	✓	✓				✓	✓	✓				✓	
**Inorganic pigments**													
Orpiment	✓							✓	✓			✓	
**Mordants**													
Green vitriol												✓	✓

✓ indicates the presence of the substance within the sampling point; for clarity, “INV_X_” (X = 11, 13, 15, or 20) was omitted from the sampling points’ acronyms.

Traces of Sulphur (S) and manganese (Mn) were observed on the wallpapers’ verso, although S was solely recognized in the not-written areas, except for INV_15, while a higher distribution of Ca and iron (Fe) was seen in this side as compared to the recto (Fig. 6a, b, Supplementary Fig. 4a). Since these two elements, along with lead (Pb), were preponderant in the written portions of INV_11 and INV_13 (Fig. 6b, Supplementary Fig. 4a), they may be components of the ink (e.g., iron gall ink) used for the selvages^12^, which are a trademark of *Kinkarakawa-gami*^1^. This conclusion cannot be drawn for INV_15_2 and INV_20_2, due to the strong presence of arsenic (As) and mercury (Hg) (Supplementary Fig. 4a), deriving from the coloured areas of the recto (Fig. 6c, Supplementary Fig. 5b) and/or a metal diffusion from one overlapping wallpaper to another, which may have hidden Ca and Fe signals.

### Gilding of the paper with metals

Tin (Sn) was ubiquitously present in the wallpapers, revealing a greater signal distribution on their recto than their verso (Fig. 6), which is coherent with the gilding technique used by Japanese artisans, who covered the paper surface with a lacquer mordant and a metal foil (i.e., silver, gold or tin) and adhered to the embossed pattern^7^. In this regard, CLSM imaging of the wallpapers’ recto showed an amorphous and heterogeneous surface attributable to the artefact’s pattern, the inorganic pigments, and/or organic pigments (Fig. 5b). To better validate whether the wallpapers featured a multilayer structure as described in the literature^1,2^, a 3D reconstruction of a portion of INV_15 recto only partially covered by the motif pigmentation was performed (Supplementary Video 2). As a result, two distinct layers were detected: the top layer displayed the amorphous structure characterising the pigmentation, while the bottom layer featured an underlying matrix of fibres resembling that of the verso (Fig. 5c, d, Supplementary Video 2). Since the gilding practice was first carried out by Rottmann, Strome & Co factory in Yokohama (1882) yet abandoned from 1890 onwards^2^, the presence of multilayers containing Sn within the wallpapers suggested their belonging to the second period of *Kinkarakawa-gami* production.

### Kinkarakawa-gami’s decoration

Despite the published literature on *Kinkarakawa-gami* art, indications regarding inorganic and organic pigments, as well as binders used for artefacts’ decoration is not reported yet, hence constituting a gap to be filled to enable their conservation-restoration.

#### Inorganic pigments

XRF spectroscopy revealed the recurrent presence in the artefacts of Si, S, Fe, Mn, Zn, As, whereas Hg, copper (Cu), chromium (Cr), and Pb were detected in specific coloured portions (Fig. 6), indicating the use of several inorganic pigments for the decoration. Although most of these pigments have strong IR contributions below 600 cm^−1^^13^, some absorption bands attributable to the yellow pigment orpiment (As_2_S_3_, *Sekio*; 1035, 913, and 820 cm^−1^) and its degradation (-AsOFe stretching mode 830–840 cm^−1^) were frequently found within the µATR-FTIR spectra (Table 2, Supplementary Table 1–4), agreeing with the detection of As and S through XRF spectroscopy (Fig. 6). Since up to 1885 the wallpaper production did not involve As-compounds^2,6^, these results suggested artefacts’ manufacture between 1886 and 1889, which is a rather tight timeline unlikely to be achieved through other techniques (e.g., Accelerator Mass Spectroscopy (AMS)-radiocarbon dating featuring ± 20 years of experimental error)^14^.

Fe ubiquity on the recto’s background (Supplementary Fig. 4b) may instead indicate the application of natural iron oxide pigments, such as yellow—*Odo*—, brown—*Taisha*—and red—*Kincha*—ochres^15^, to obtain gold-bronze shades, which is another distinctive feature of this Japanese art^7^. Moreover, these pigments contain silicates and manganese dioxide (MnO_2_) as either components or impurities^16^, supporting the frequent detection of Si and Mn in traces (Fig. 6). An additional trace element observed in INV_11_7 and INV_13_3 was S (Supplementary Fig. 4b), whose contribution might rely on the use of tin sulphide (SnS_2_), which was known in Asia since 300 BC^17^, as a bronze-gold pigment for these backgrounds. The variability of the elemental composition in recto’s backgrounds was inferred by the detection of Zn (INV_11_5, INV_11_7, and INV_15_3), As (INV_15_3 and INV_20_5), Pb (INV_11_5), or Hg (INV_15_3; Fig. 6c). In this regard, the simultaneous presence of S and Hg (INV_15_3), or S and As (INV_20_5; Supplementary Fig. 4b) suggested that red mercury- (HgS; cinnabar or *Shu*) and yellow/red arsenic-sulphides (*Sekio* or red realgar—*Yuo*, AsS or As_4_S_4_) were the main pigments of INV_15 and INV_20 recto’s backgrounds^15^. As and Zn traces in INV_15_3 could be also linked to *Shu*, which generally contains these elements as impurities^17^. Furthermore, Zn-based paint driers may have been applied to favour the pigments’ deposition and solidification on the tin foil^18^, while Chinese white (zinc oxide; ZnO) and zinc sulphide (ZnS) could be used as white pigments^19^. To improve the brightness of the latter, artisans even mixed them with lead white [basic lead carbonate; 2PbCO_3_·Pb(OH)_2_]^20^, justifying the Pb contribution observed in INV_11_5 (Supplementary Fig. 4b).

The painted areas of the wallpapers’ recto were deeply analysed focusing on the most representative colorations (i.e., red, green, brown, and black). Similar chemical compositions were observed for the red colour in INV_11_6 and INV_15_4, where the featuring elements were Hg and S, indicating the utilization of *Shu*^15,17^, while traces of Si were detected only in INV_11_6 (Fig. 6d, Supplementary Fig. 5a). Moreover, the large signal of Hg observed in both INV_11_6 and INV_15_4 might be responsible for the Sn underestimation, as the high atomic number of Hg can mask Sn contribution. The green-coloured areas of INV_11, INV_13, and INV_20 displayed instead a high variability in elemental composition (Fig. 6e), inferring that these portions could contain multiple pigments. Particularly, Pb was present only in INV_11, whose great variability in composition was further indicated by the detection of Cr and Cu (INV_11_3; Supplementary Fig. 5b), typical components of green pigments, as chromium oxide (CrO_2_), malachite green [Cu_2_(CO_3_)(OH)_2_], Verdigris [Cu_2_(CH_3_COO)2Cu(OH)_2_)], Scheele green (a mixture of CuHAsO_3_ and CuAs_2_O_3_), or emerald green [Cu(CH_3_COO)_2_3Cu(AsO_2_)_2_]^17^. The presence of Pb may be instead ascribed to (1) lead white for the outline of the leaves^15^, (2) lead–tin yellow^21^, or (3) yellow lead oxide (PbO)^22^ to achieve diverse green shades. Regardless, all the green areas exhibited S and As, whose distribution varied depending on the different colour shades, as noted in INV_11_3, INV_11_9, INV_13_4, and INV_13_8 (Supplementary Fig. 5b). Thus, *Sekio*^15^ seemed to be constantly present in the green areas^23^, suggesting (1) a combined use of Verdigris, Scheele green, or emerald green with sulphide compounds in INV_11_3 to improve both brightness and durability of the pigments^24^, as well as (2) the addition of *Sekio* to an organic dark (blue/purple) pigment to obtain the green colour^15,25,26^. Except for INV_20, the intense Fe signal characterising the recto’s green-painted sections (Fig. 6e) could imply the combination of green/yellow inorganic pigments with natural ochres^15^, as also supported by the detection of Si, Mn, and Zn in traces (Fig. 6e, Supplementary Fig. 5b). Nevertheless, µATR-FTIR spectra of INV_20_3 exhibited –SO_4_ asymmetric (1120–1040 cm^−1^ and 827 cm^−1^) and symmetric (983 cm^−1^) stretching, –FeOSOFe vibration (955 cm^−1^), as well as –FeO stretching (830 cm^−1^) characteristic of the Fe-based mordant green vitriol (FeSO_4_·xH_2_O) (Table 2, Supplementary Table 4), which was likely used in this wallpaper. Several brown drawings were part of INV_11 and INV_13 (Table 1), where Fe detection (Fig. 6f, Supplementary Fig. 5c) might infer to the utilization of *Taisha*^7^, also supported by the low content of Si, Mn, and Zn, which confer diverse yellow–brown and red-brown shades to the pigment^16^. Additionally, lead white and/or lead oxide^15,22^ could be present in INV_11_4 and INV_13_5, as indicated by Pb traces, while INV_13_6 and INV_13_9 showed a relatively high distribution of both As and S (Fig. 6f) attributable to the addition of *Sekio* or *Yuo* to get unique brown shades^23,26^. Finally, the black colour was only present in INV_11_10, whose elemental analysis revealed traces of S and the three ubiquitous elements Ca, Fe, and Sn (Fig. 6g), implying the use of an organic black pigment for this colour^15^.

#### Organic pigments

Several IR absorption bands typical of the natural purple-pigment Indigo were detected in the green areas of INV_11_3 and INV_13_8 (Fig. 2a, b, Table 2, Supplementary Table 1–2), whose similarity was confirmed by the partitioning of their spectra in cluster 4 (average s_i_ = 0.56; first interval) or 2 (average s_i_ = 0.70; second interval), as well as their PC2 values (Figs. 3c–e, 4c–e). This observation agrees with the practice of Japanese artists, from the late Edo period (1840–1860) onwards, to mix Indigo with lime, ashes, and *Sekio* as an alternative to malachite green^16,25,26^. On the other hand, the INV_20_3 spectrum, belonging to cluster 1 for both the first (average s_i_ = 0.83) and second (average s_i_ = 0.76) intervals (Figs. 3c–e, 4c–e), suggested that diverse techniques could have been used to obtain the green colour in INV_20 as compared to INV_11 and INV_13. Although INV_20_3 featured some Indigo’s IR contributions, the detection of vibrational modes typical of -SO_4_ and -FeO groups (Supplementary Table 4) indicated that iron sulphate mordants, such as green vitriol, were supplied to Indigo and *Sekio* obtaining the green colour. Indeed, a similar procedure was developed by English artisans, who added pearl ashes, lime, *Sekio*, and green vitriol to Indigo syrup, favouring both the precipitation of Fe protoxide by the lime, as well as the deoxygenation of Indigo, which acquired a dark green shade^27,28^. Besides, only a few of Indigo’s IR contributions were identified in red and brown drawings (Table 1, Supplementary Tables 1–2, 4), reasonably due to (1) Indigo diffusion from adjacent areas containing the organic pigment or (2) its utilization to obtain diverse colour shades. The µATR-FTIR spectra collected for brown and red drawings of INV_11 and INV_13 (Table 1) showed a high degree of similarity, as they all clustered with INV_11_3 and INV_13_8 spectra for both the 3770–2750 cm^−1^ and 1840–719 cm^−1^ intervals (Figs. 3c–e, 4c–e). INV_13_9 constituted the only exception, as it was grouped in cluster 1 (Figs. 3c, 4c), underlining that diverse organic substances, alongside the inorganic *Sekio* (Supplementary Fig. 5c), may be used for this brown sampling point. Since Indigo features low IR contributions in the 3770–2750 cm^−1^ interval^29^, indications regarding its presence within red and brown drawings can be better obtained by focusing on the MBD-based analysis of the second IR interval, where INV_13_5.1 was the deepest curve (Fig. 4f), while INV_11_6, INV_15_4, and INV_20_4 were the most external and variable of the cluster (Fig. 4d, Supplementary Fig. 3b). Hence, it is reasonable to suggest that Indigo diffusion was responsible for IR contributions in INV_11_6 and INV_20_4, while it was likely used for INV_11_4, INV_13_3, and INV_13_5, being part of the core cluster (Supplementary Fig. 3b). Moreover, INV_15_4 partitioning within cluster 2 for the 1840–719 cm^−1^ interval (Fig. 4c–e) may be due to the presence of other organic substances having absorption bands in common with Indigo. Indeed, any of typical IR contributions of this organic pigment were observed in INV_15_4 μATR-FTIR spectrum (Supplementary Table 3), which also featured a low depth value, as well as a high PC2 score, with respect to the other spectra clustering together (Fig. 4d, Supplementary Fig. 5b).

#### Organic binders

The identification of inorganic pigments highlighted the need for Japanese artisans to use binders (e.g., proteinaceous binders, vegetable oils, waxes, and lacquers) for their painting^15^. Although the type of these substances is generally determined through FTIR, the wallpapers’ complexity impaired their univocal identification, since they had similar IR absorption bands and their specific vibrational modes often overlapped with each other (Supplementary Tables 1–4)^30,31^. In this regard, the clustering analysis in the 1840–719 cm^−1^ interval revealed that spectra featuring Indigo’s IR contributions grouped in cluster 2, while cluster 1 featured the verso spectra and those where the organic pigment was absent or less present, in which overlapping vibrational modes of cellulose, lignin, silicates, carbonates, and organic binders were ubiquitous (Fig. 4c, Table 2). Similarly, although four clusters were identified for the µATR-FTIR spectra in the 3770–2750 cm^−1^ interval (Fig. 3c–e), several absorption bands attributable to these substances were detected (Supplementary Tables 1–4). Nevertheless, the type of organic substances was presumed, when possible, by comparing their IR absorption bands with those characterising the organic binders (Supplementary Tables 1–4). Thus, vibrational modes typical of proteinaceous binders (INV_15) or waxes (INV_13_9 and INV_20) were detected (Supplementary Tables 3–4), which is in agreement with the Japanese traditional use of animal glue (*nikawa*), gelatine cubes, and beeswax as binders^15,25^. Since IR absorption bands distinctive of proteinaceous binders or waxes are more present in the 3770–2750 cm^−1^ interval^30,31^, the partitioning of µATR-FTIR spectra of INV_15 recto in cluster 3 (average s_i_ = 0.66), INV_13_9 and INV_20_3 in cluster 1 (Fig. 3c, d) statistically supported these observations. Particularly, the MBD-based analysis of cluster 1 revealed a high degree of similarity between these µATR-FTIR spectra, representing both the deepest curve (INV_13_9) and the core (70%) of the cluster itself, while INV_11_4.1 was the most external spectrum (Fig. 3f, Supplementary Fig. 2a), where IR absorption bands typical of waxes were not detected (Supplementary Table 1). Moreover, although vibrational modes characterising waxes are less present in the 1840–719 cm^−1^ interval^30^, the comparable PC2 values of INV_13_9, INV_13_9.1, INV_13_9.2, and INV_20_3 spectra highlighted their higher variability, yet intrinsic similarity, than other curves belonging to cluster 1(Fig. 4d).

Several µATR-FTIR spectra displayed also vibrational modes distinctive of organic vegetable lacquers (Table 2, Supplementary Tables 1–4), which grouped in cluster 3 in the 3770–2750 cm^−1^ interval (Fig. 3c–e). In this regard, the so-called lacquering procedure for the wallpapers’ production involved the mixing of plant-extracted lacquers—likely the *Urushi* one, based on the Japanese tradition^32^—with *Odo*, *Taisha*, and *Kincha* to confer the yellow-gold background characteristic of the *Kinkarakawa-gami*’s recto^7^. Indeed, the *Urushi* lacquer features a strong IR contribution around 3400 cm^−1^ attributable to –OH stretching^32^, which, alongside the vibrational modes of proteinaceous binders, was a distinctive trait of the µATR-FTIR spectra belonging to cluster 3 (first interval; Fig. 3c–e). In line with this, INV_15_4 resulted the deepest curve identified for this cluster (Fig. 3f), while INV_13_5, where the –OH stretching of the *Urushi* lacquer was absent (Supplementary Table 2), was the most external one (Fig. 3d, Supplementary Fig. 2a).

### Isolation and identification of microorganisms populating the wallpapers

A microbiological evaluation of specific areas of these artefacts was carried out to assess the role of microorganisms in either the degradation or damage prevention of the wallpapers, as well as the hindrance in identifying IR contribution of organic substances due to the interference derived from macromolecules’ vibrational modes^33,34^. A wide array of microorganisms populates different works of art, as their organic and inorganic substances constitute carbon and essential element sources for bacteria and fungi^34^. The microbial presence on artistic items is also favoured by environmental factors (e.g., low ventilation, high humidity) and the poor state of conservation of the artefacts, which allows the microbial growth under oligotrophic conditions^34^. Particularly, microorganisms tend to irreversibly attach to the artefact surface, forming communities defined as a biofilm, where its complex hydrogel matrix—made of proteins, lipids, and polysaccharides—confers to bacteria and fungi protection from external factors^35^. Additionally, less than 10% of microorganisms populating different niches can be cultured through standard procedures^34^, making it almost impossible to entirely identify the microbial community associated with artistic items. Several bacteria and fungi (Table 3) were isolated from sampling points of INV_11, INV_13, and INV_15, while any cultivable microorganism was not retrieved from INV_20. Bacterial isolates were identified as part of the Firmicutes (i.e., *Bacillus coreaensis*, *B. pocheonensis*, *B. onubensis*, *Staphylococcus capitis*, and *S. epidermidis*) and Actinobacteria [i.e., *Kocuria rizophila* (former *Micrococcus luteus*) and *Micromonospora chokoriensis*] phyla, while all the detected fungi belonged to the Ascomycetes phylum, although four genera were observed (Table 3). These results are in line with the studies reporting the isolation of cultivable microorganisms from wall or easel paintings^34,36^.

**Table 3:** Identification of bacterial and fungal strains isolated from the four *Kinkarakawa-gami* analysed.

Bacterial isolates	Identity (%)	Type reference strains	Accession number	Taxonomy
C_4_(11)3	99	*Bacillus coreaensis*	JN578481	Firmicutes
C_4_(13)1	94	*Bacillus pocheonensis*	AB245377	
C_4_(13)2	99	*Bacillus onubensis*	NSEB01000017	
C_2_(11)2	80	*Staphylococcus capitis*	L37599	
C_5_(13)2	97	*Staphylococcus epidermidis*	UHDF01000003	
C_4_(15)1				
C_7_(11)1	99	*Micromonospora chokoriensis*	LT607409	Actinobacteria
C_2_(11)1	98	*Kocuria rizophila*	NCTC 2665	
C_5_(13)1	95		CP001628	
C_4_(11)1	99	*Hirsutella* sp. B YA-2015	KJ524675	Ascomycetes
C_4_(15)2	98	*Penicillium georgiense*	KX664381	
C_4_(15)3	99	*Aspergillus costaricaensis*	MK910049	
C_4_(11)2	98	*Cladosporium cf. ramotenellum*	KY781778	

C_*n*_(*X*)*m*: C_*n*_ indicates the chosen sampling point, as depicted in Fig. 1; *X* the catalogue number of the wallpaper from which the bacteria and/or fungi were isolated; *m* represents the number of bacterial or fungal isolates investigated.

The microbial isolates C_4_(11)3, C_4_(13)1, C_7_(11)1, and C_4_(11)2 closely related to *B. coreaensis*, *B. pocheonensis*, *M. chokoriensis*, and *Cladosporium cf. ramotenellum* respectively (Table 3), and autochthonous of *Kinkarakawa-gami*, are commonly found in several environmental matrices of Korea, China, Thailand, and Japan^37–40^, confirming the eastern provenance of these wallpapers. Besides, these microorganisms hold enzymatic assets (i.e., xylanases and laccases) that enable them to degrade and depolymerize (1) lignin and cellulose compounds^37,40–44^, (2) lacquers^45–47^, and (3) organic pigments^46,48^. Indeed, *Bacillus*, *Staphylococcus*, and *Micrococcus* spp. produce extracellular xylanases^42,43,49^, which efficiently hydrolyse lignocellulosic material and the Indigo pigment^42,43^, a biotic aspect that supports their presence on INV_11_1, INV_11_4, INV_13_5, and INV_13_4 (Table 3). Laccases are instead Cu-polyphenol oxidases firstly identified in the exudates of the Japanese *Rhus verniczfera* plant^50^ from which the *Urushi* lacquer is extracted^32^, yet also recognized as enzymatic catalyst of most fungal strains^47^. Since these enzymes are responsible for the degradation of phenol substrates^45,47^, the partial identification of vibrational modes typical of urushiol polymerization or Indigo (Supplementary Tables 1–4) could be ascribed to the biotic deterioration of both *Urushi* lacquer and organic pigment, as Japanese lacquer IR contributions were found within sampling points where fungal strains were isolated (Tables 2, 3). A similar conclusion can be made for C_4_(11)3 and C_4_(11)2 isolates phylogenetically related to *B. coreaensis* and *C. ramotenellum* respectively, whose production and secretion of xylanases and laccases is one of their distinctive metabolic traits^37,40,44^. Further, *Bacillus* [C_4_(13)2], *Staphylococci* [C_5_(13)2 and C_4_(15)1)], and *Micrococci* [C_2_(11)1 and C_5_(13)1] species are among the most persistent strains capable of metabolizing honey and beeswax^51^, while *M. chokoriensis* [C_7_(11)1] and *C. ramotenellum* [C_4_(11)2] are proficient in degrading gelatine and starch, which were two of the most used binders in Japanese tradition^15,27^.

The presence of microorganisms can also be linked to their tolerance and/or resistance towards a broad spectrum of metal and metalloid compounds^52^, which are the main components of inorganic pigments. For instance, bacteria belonging to the Firmicutes and Actinobacteria phyla can detoxify their surrounding environment from toxic metals or metalloids, even using them as a terminal electron acceptor to produce energy^52,53^. *Bacillus*, *Staphylococcus*, and *Micrococcus* spp. are overall present in paintings where carbonates (chalk—CaCO_3_) and silicates (quartz—SiO_2_) are abundant^54,55^, due to their ability to overcome the challenge deriving from these minerals. *Bacillus* spp. are also highly tolerant towards Pb-^56^ and Fe-containing compounds^57^, being able to transform lead acetate [Pb(CH_3_CO_2_)_2_]^56^, hematite (Fe_2_O_3_) and its hydrated forms^58^, as well as to oxidize Fe(II) to Fe(III), as in the case of *B. pocheonensis*^59^. Analogously, *S. epidermidis*, *M. chokoriensis*, and *K. rizophila* strains were studied for their resistance against Pb-containing compounds^60–62^, while the fungal strain *Hirsutella* spp. showed tolerance to Ca- and Fe-sulphates^63^. Thus, the elemental composition of INV_11 and INV_13 (Fig. 6) justified the presence of microorganisms (Table 3) that hold metabolic traits allowing them to survive on metal-rich wallpapers. Although INV_15_4 displayed a high amount of *Shu* (HgS) (Supplementary Fig. 5a), one bacterial [C_4_(15)1] and two fungal [C_4_(15)2 and C_4_(15)3] strains related to *S. epidermidis*, *Penicillium georgiense*, and *Aspergillus costaricaensis* were isolated (Table 3), highlighting their great tolerance towards Hg-containing compounds^64,65^. Indeed, these fungal strains were responsible, among others, for the darkening of cinnabar (*Shu*), as reported elsewhere^66^. The isolation of *S. epidermidis* and *Hirsutella* strains could also derive from anthropogenic/environmental contamination, as they are human and nematode pathogens respectively^63,64^, reflecting the artefacts’ poor state of conservation.

Besides, the local heterotrophic microflora (i.e., *Bacillus, Micromonospora*, and *Micrococcus* species) can produce secondary metabolites with antimicrobial properties as a defence mechanism under stress conditions^34,67–69^. Thus, Firmicutes and Actinobacteria could act as biocontrol agents for the preservation of cultural heritage^34^. These observations, along with the advanced state of deterioration that was macroscopically visible for INV_20, may indicate the key role played by bacteria belonging to *Bacillus, Micromonospora,* and *Micrococcus* genera in controlling and preventing the further degradation of the colonized wallpapers; indeed, INV_20 was the only artefact from which any cultivable microorganism was not isolated.

## Conclusion

This multidisciplinary study allowed to unveil physical–chemical features regarding the composition, manufacture, and dating of the collectively important cultural heritage represented by *Kinkarakawa-gami* works of art. The experimental evidence gathered was corroborated through the uncommon yet resourceful and innovative *in blind* functional data statistical analysis. Indeed, given the complexity of the studied wallpapers in terms of IR vibrational modes ascribed to (1) substances used for their fabrication, (2 physical–chemical degradation products, and (3) the presence of a microbiota, this statistical approach has proved greatly helpful to identify and confirm trends, differences, and similarities observed among the four *Kinkarakawa-gami* artefacts. Microbiological investigations supported the Eastern provenance of these wallpapers, however, whether the cultivable microbes act as deteriogen or biocontrol agents is yet to be defined; hence, DNA sequencing-based technology (e.g., study of the microbiome) represents the new frontier to unveil the identity of uncultivable microorganisms and, alongside physical–chemical characterisation, will improve the development of innovative, promising, and *eco-friendly* restoration strategies for the conservation of cultural heritage.

## Experimental section

### Materials

The four wallpapers here studied belong to the V. Ragusa-O’T. Kiyohara collection of Palermo (Italy). Given the complexity and richness of these artefacts in terms of details and depicted colours, an extensive sampling of the wallpapers (Table 1) was performed to thoroughly analyse them.

Tryptic soy, malt extract, and agar technical were purchased from Sigma-Aldrich® (Milan, Italy), while QIAquick PCR purification kit was obtained from QIAGEN (Milan, Italy).

### X-Ray fluorescence (XRF) spectroscopy

A Tracer III sd Bruker AXS (Bruker, UK) equipped with Rhode anode and working at 40 kV and 11 µA was exploited for XRF analyses, whose acquisition time was 30 s. Element identification and XRF spectra analysis was performed by using ARTAX® software, which was provided with the instrument, while R 3.6.1 and OriginPro® 2016 software were used for the representation. Elemental compositions of wallpaper sampling points are reported in terms of Net Area (× 10^4^ a.u.), which represents the integral intensity of X-ray emission characteristic of each element obtained after performing the Bayes deconvolution and deduction of the background intensity^70^. These results are to be considered as a qualitative estimation regarding the presence and abundance of diverse elements within the chosen sampling points, as any appropriate standard was not used to determine the concentration of each element.

### Attenuated total reflectance-Fourier transform infrared (ATR-FTIR) spectroscopy

ATR-FTIR spectra were recorder by using a µFTIR Lumos (Bruker, UK) equipped with a Platinum ATR and an IR microscope featuring 0.1 µm as lateral resolution. The spectra were collected in the 4000–600 cm^−1^ range, with a resolution of 2 cm^−1^ and 60 scans *per* each sampling point, and they were subsequently analysed through the software OPUS(7.5)®, which was provided with the instrument, as well as OriginPro® 2016.

### Statistical analyses of µATR-FTIR spectra

Based on the structure of µATR-FTIR spectra, they can be assimilated to complex sets of data (i.e., functions, where each spectrum corresponds to a distinct function) varying over a continuum (i.e., wavenumber range) that, taken together, can be considered as single curves. Hence, *in blind* functional data analysis (FDA) was applied as the statistical methodology on these spectra^71^ that were split, based on the wavenumber range acquisition, in 3770–2750 cm^−1^ and 1840–719 cm^−1^ intervals, which were singularly analysed by performing a hierarchical clustering of µATR-FTIR curves with the respect to their shape. The quantification of each curve’s cohesion to its own cluster as compared to the separation from the other clusters was obtained through a silhouette (s_i_) measurement, while the optimal number of clusters was determined by maximizing the average s_i_ to the configuration obtained in the hierarchical clustering. Functional Principal Component Analysis (FPCA) was then performed to derive the PCs inside the final clusters^71^ and find an optimal orthogonal linear projection of the curves on a d-dimensional subspace (R^d^ when d = 2 or d = 3) to minimize the expected value of the squared error due to the projection. Lastly, a data depth algorithm based on the Modified Band Depth (MBD) was constructed focusing on centrality and separation of the spectra^72^, allowing to identify both the most representative and the most external curves of each cluster. All the statistical analyses were performed by using the R 3.6.1 software; a more extensive review of the performed statistical analyses and the R-packages used is reported in the Supplementary Methods section.

### Fluorescence microscopy

Fragments of the wallpapers were imaged both on recto and verso sides by a Leica TCS SP5 fluorescence confocal laser scanning microscope (CLSM), using a 40X-1.25 NA objective (Leica Microsystems, Germany). Images were acquired under two-photon excitation at 830 nm in the 450–700 nm emission range. The samples were soaked in glycerol during the imaging procedure. The same setup was applied for a 3D reconstruction of the wallpaper multilayers. The data were analysed by ImageJ software.

### Microbiological analyses

The sample areas of interest of *Kinkarakawa-gami* were gently swiped with sterile cotton swabs, which were then suspended in the physiological solution (sodium chloride 0.9% w/v) for 30 min. Afterward, the suspensions were serially diluted, being aliquots (100 μL) spread onto both tryptic soy and malt extract agar plates to isolate either bacterial or fungal strains respectively, whose biomass growth was carried out at 30 °C for 5 days under static conditions.

Polymerase chain reaction (PCR) was performed (Supplementary Methods), following thermocycler conditions described elsewhere^73^, on the extracted and purified genomic DNA to obtain the 16S rRNA gene product and the internal transcribed spacer (ITS) region in the ribosomal RNA operon. To identify—at genus level—the isolates retrieved from *Kinkarakawa-gami*’s art, PCR products were purified through QIAquick PCR purification kit, according to the manufacture’s protocol, sequenced (BMR Genomics, Padova, Italy), and searched for nucleotide homology with other microorganisms (Supplementary Methods).

## Supplementary information

Supplementary Information 1.

Supplementary Movie 1.

Supplementary Movie 2.
