# Supplementary material for: A combined physical–chemical and microbiological approach to unveil the fabrication, provenance, and state of conservation of the Kinkarakawa-gami art
Source: Sci Rep. 2020 Oct 2;10:16072. doi: 10.1038/s41598-020-73226-6 (PMC7532529; doi:10.1038/s41598-020-73226-6)
Supplement: Supplementary file 1 — Supplementary Information 1. [file 41598_2020_73226_MOESM1_ESM.docx]

Supplementary information for:

**A combined physical-chemical and microbiological approach to unveil the fabrication, provenance, and state of conservation of the *Kinkarakawa-gami* art**

Elena Piacenza^1,2^, Alessandro Presentato^2,*^, Francesca Di Salvo^3^, Rosa Alduina^2^, Vittorio Ferrara^1,4^, Valeria Minore^2^, Antonio Giannusa^4^, Giuseppe Sancataldo^5^, Delia Francesca Chillura Martino^1,2,*^

^1^National Interuniversity Consortium of Materials Science and Technology (INSTM) – UdR of Palermo, 50121, Florence, Italy

^2^Department of Biological, Chemical and Pharmaceutical Sciences and Technologies (STEBICEF), University of Palermo, 90128, Palermo, Italy

^3^Department of Agricultural, Food and Forest Sciences, University of Palermo, 90128, Palermo, Italy

^4^*Vincenzo Ragusa and O’Tama Kiyohara* museum, Liceo Artistico Statale Vincenzo Ragusa e O’Tama Kiyohara, 90129, Palermo, Italy

^5^Department of Physics and Chemistry (DIFC) Emilio Segrè, University of Palermo, 90128, Palermo, Italy

^*^Corresponding authors:

Alessandro Presentato, PhD, Researcher, Department of Biological, Chemical and Pharmaceutical Sciences and Technologies (STEBICEF), University of Palermo, Viale delle Scienze 17, Palermo, 90128, Italy; phone number: +3909123897306; email: alessandro.presentato@unipa.it

Delia Francesca Chillura Martino, PhD, Associate Professor, Department of Biological, Chemical and Pharmaceutical Sciences and Technologies (STEBICEF), University of Palermo, Viale delle Scienze 17, Palermo, 90128, Italy; phone number: +3909123897981; email: delia.chilluramartino@unipa.it

**Historical information on the Japanese museum “Vincenzo Ragusa” of Palermo**

Subsequently to the first relations that the “Land of the Rising Sun” had with the West from the XVI century, due to Christian religious orders – unsuccessfully trying to evangelize this country – Japanese borders soon closed to reopen in the middle of XIX century in response to American and European pressures. Three Italian artists were invited to found the western art school Kobu Bijutsu Gakko in Tokyo, while a massive interest towards oriental prints and artistic objects started to diffuse in the West, leading *Japonisme* to spread and somehow influence the western artistic culture of that time. Among the artists sent to Japan, the sculptor Vincenzo Ragusa acted as a link between the two intrinsically diverse Sicilian and Japanese cultures with both his personal artistic experience and his marriage with the sensitive Eastern painter O’Tama Kiyohara, who lived in Palermo for 51 years and, under Ragusa’s guidance, managed to master the western expressive ways that were before unknown to her. In 1884, these two artists founded the oriental art school holding the first Japanese museum in Sicily, exhibiting several Japanese handicrafts purchased there by the sculptor, as well as later artistic additions, most of which were faithfully reproduced by O’Tama Kiyohara as watercolour tables preserved between Tokyo and Palermo. Since the museum and workshops focused on oriental techniques of the school were abolished after a few years from their foundation by a ministerial disposition, Ragusa was forced to sell the Collection to the Pigorini Museum in Rome. Nevertheless, precious fabrics, some ceramic and lacquer bowls, the magnificent watercolours painted by Kiyohara, as well as several unique *Kinkarakawa-gami* wallpapers remain on display in today’s *Vincenzo Ragusa and O’Tama Kiyohara* museum, as evidence of Ragusa’s original artistic will.

**Supplementary Methods**

***Statistical analyses of µATR-FTIR spectra***

The structure of the µATR-FTIR spectra allows their representation as a realization of a univariate functional random field. In the final spectrum, the couple [w, y(w)] denotes the wavenumber and the observed intensity of the i^th^ spectrum for w **∈** W = [4000-400] cm^-1^ and i = 1,2,…n (where n is the number of obtained spectra), respectively; the resulting functions are treated as elements of an infinite-dimensional Hilbert space. Thus, by using the Hilbert structure of the function spaces resulting from the L^2^ norm, the functional data analysis (FDA) estimates the underlying function itself f(w) on the basis of the y(w) values (i.e., measured intensities in the spectra) observed in correspondence of the sampled w_i_ of the wavenumber domain. The estimation procedure is based on a penalized cubic regression spline [1]. Practically, each of the 21 analysed curves was estimated through a linear combination of B-spline functions of order 3 with equidistant knots; the generalized cross validation (GCV) criterion is implemented for selecting the number of parameters of the model, while the coefficients were determined by minimizing the sum of least squares distances with a penalization defined in terms of second derivatives.

The cluster analysis of curves is performed for grouping µATR-FTIR spectra with similar shapes in homogeneous categories. The basic idea is to divide the estimated spectra into groups (clusters) such that the shape variability is small within the clusters and as large as possible across them. The quantification of shape variability is based on the L^2^ norm of the Hilbert space. In clustering homogeneous curves with respect their shape, two sets of unknowns needed to be determined: the number of clusters *k* and the matrix *M* assessing the allocation of the elements to the *k* clusters, denoted as C_j_ for ij=1, 2…k. The hierarchical clustering was implemented with an agglomerative algorithm [2], which allowed to determine the shape clustering by simultaneously checking the relative locations of the IR absorption bands and their wavenumber range, discarding a cluster configuration if the range was too large. Subsequently, silhouettes were measured, following equation 1:

$$s_{(i)}=\frac{b_{\left( i \right)}-a_{(i)}}{{max}_{(i)}\{a_{\left( i \right)},b_{\left( i \right)}\}} (eq.1)$$

Where a_(i)_ and b_(i)_ are the mean distances between the i^th^ curve and the others of the same or different cluster, respectively; in the range from −1 to +1, high value of s_(i)_ indicates that the object is well matched to its own cluster, and it is an indication of the appropriateness of the obtained clustering configuration. After a configuration is selected, the centrality of a µATR-FTIR spectrum within a cluster of spectra and their arrangement by their degree of centrality in a natural centred-outward order can be obtained. A proper tool to analyse this aspect is the statistical depth that generalizes order statistics (i.e., ranks and medians) to higher dimensions and functional data, which are here represented by µATR-FTIR spectra. Hence, the Modified Band Depth (MBD) function was used to assess an order from centre (highest depth) outwards (lowest depth), and an algorithm was proposed to also allow the identification of a representative (or deepest) curve, based on the most central spectra, as well as the most external curves. The obtained results explained the outlining of µATR-FTIR spectra in terms of different shape, as the IR absorption bands were homogenously located inside the clusters. For all the above-mentioned statistical analyses, *fda*, *cluster*, *dedextend*, and *depthTools* R-packages [3-6] were used.

***Microbiological analyses***

The bacterial and/or fungal genomic DNA were extracted and purified through the chloroform-phenol method applied on the recovered biomasses grown for 24 hours at 30 °C with shaking (150 rpm) in the liquid-rich tryptic soy or malt extract broth, respectively. The universal pair primers F1/R12 (for bacteria) [7] and ITS1(F)/ITS4(R) (for fungi) [8] were used for the PCR reaction, while the final sequence outputs were searched for nucleotide homology using EZ Taxon-E [9] and BLASTN [10] databases.

**Supplementary Table 1:** µATR-FTIR absorption bands of INV_11 and their attribution.

| **Wavenumber (cm^-1^)** | | | | **Vibrational modes** | **Compounds** |
| --- | --- | --- | --- | --- | --- |
| **INV_11_3** | **INV_11_4** | **INV_11_6** | **INV_11_7** |  |  |
|  |  |  | 3525 | ν OH | Carbonates [11] |
| 3401 |  |  | 3401 | ν OH | *Urushi* lacquer [12-13] |
|  | 3357 |  |  | ν OH | Cellulose [14]; microbial polysaccharides and proteins [15] |
| 3275 |  | 3272 |  | ν NH | Microbial proteins [15]; Indigo [16]; proteinaceous binders [17] |
| 2923 | 2921 | 2919 | 2924 | ν_as_ CH_2_ | Cellulose, lignin [14]; microbial lipids and polysaccharides [15]; binders [17-19] |
| 2853 | 2844 | 2848 | 2852 | ν_s_ CH_2_ | Microbial lipids and polysaccharides [15]; binders [17-19] |
| 1702 | 1708 | 1703 | 1706 | ν + δ SiO; ν C=O | Silicates [11], free fatty acids from waxes [20] |
| 1653 | 1653 | 1653 | 1653 | ν C=O (also Amide I);  ν *cis* HC=CH;  δ OH (water) | Microbial α-helix proteins [15]; proteinaceous binders and oils [17]; lignin [21] |
| 1626 |  |  |  | ν C=O; β OH (water)  β NH_2_ Amide II; | *Urushi* lacquer [12-13]; Indigo [16]; proteinaceous binders [17]; microbial primary amines [22] |
| 1614 |  |  |  | ν_ip_ C-C_6ring_ | Indigo [16] |
| 1585 |  |  |  | ν_ip_ C-C_6ring_ | Indigo [16] |
| 1481 | 1481 |  |  | ν C-C_6ring_; ρ_s(iph)_ CH | Indigo [16] |
| 1460 | 1456 | 1458 | 1454 | β scissoring CH_2_;  ν C-C_6ring_; ρ_as(oph)_ CH; | Binders [12-13,18-19] lignin [14]; microbial lipids [15]; Indigo [16] |
| 1437 | 1440 | 1436 | 1441 | ν_(v3)_ CO_3_^2-^;  δ_as_ (CH)CH_3_; δ_s_ CH_2_ | binders [17-19]; carbonates [23] |
| 1410 | 1411 |  |  | ν CN; ρ NH | Indigo [16]; proteinaceous binders [17] |
| 1374 | 1376 | 1373 | 1370 | δ_s_ CH_2_; δ_s_ CH | Cellulose, waxes [14]; vegetable oils [17] |
| 1317 |  | 1315 | 1317 | ω CH_2_ ν C-C_6ring_;  ν C-C_5ring_; ρ_as(oph)_ CH | Indigo [16]; Cellulose, lignin [24] |
| 1296 |  |  |  | ρ_s(iph)_ CH; β_ip_ C-C_5ring_ | Indigo [16]; binders [17-19] |
| 1257 | 1263 | 1260 | 1262 | β guaiacyl ring; β OH | Lignin [14] |
| 1195 |  |  |  | ν C-C_5ring_; ρ_as(oph)_ CH;  β_ip_ C-C_6ring_; ν CN | Indigo [16]; binders [17-19]; microbial aliphatic amines [22] |
| 1170 | 1167 | 1173 |  | ν CN; ρ_as(oph)_ CH;  ρ NH; ν C-O; ν SiO | Silicates [11]; Indigo [16]; triglyceride ester linkage in vegetable oils [17]; β_(1,3)_ glycosidic bonds [25] |
|  |  |  | 1148 | ν_as_ C-O-C | Microbial glycogen and nucleic acids [15] |
| 1105 | 1107 |  | 1105 | ν_s(v1)_ CO_3_^2-^; ν C-O;  ν_as(ip)_ glucose ring;  ν CN | Carbonates [11]; binders [17-19]; microbial aliphatic amines [22]; cellulose [24] |
|  |  | 1096 |  | ν_as_ SiO | Silicates [11] |
| 1071 | 1078 |  | 1077 | ν_as_ SiO; ν C-O; ρ CO;  ν C-C_5ring_; ν_s_ PO | Silicates [11]; binders [12-13,17]; microbial nucleic acids and phospholipids [15]; Indigo [16]; β_(1,3)_ glycosidic bonds [25] |
| 1034 | 1030 | 1030 | 1034 | ν_s_ C-O; ν_as_ SiO | Cellulose [14,26]; binders [17-19]; orpiment [18]; β_(1,3)_ glycosidic bonds [25] |
| 1009 | 1008 | 1003 |  | ν C-C_6ring_ breath._as_ | Silicates [11]; Indigo [16] |
|  | 935 | 939 |  | δ =CH | Microbial alkyl halides, carboxylic acids and amines [22]; β_(1,3)_ glycosidic bonds [25] |
| 912 | 912 | 909 | 909 | β-linkage glucose ring; β CH;ν_as_ SiO | Silicates [11]; orpiment [18]; cellulose [26] |
| 880 | 881 | 879 | 877 | ρ_op(v2)_ CO_3_^2-^; β CH;  β_ip_ C-C_6ring_; β_ip_ C-C_5ring_ | Indigo [16]; vegetable oils and waxes [17-19]; carbonates [23] |
| 820 | 825 | 826 | 821 | ρ_op(v2)_ CO_3_^2-^; β CH;  β_ip_ C-C_5,6rings_; | Indigo [16]; vegetable oils [17]; carbonates [23]; α-glycosidic linkage [25] |
|  | 800 | 801 | 805 | Iα cellulose;  ρ_op(v2)_ CO_3_^2-^ | Carbonates [11]; orpiment [18]; cellulose [26] |
| 785 | 780 | 781 | 781 | ρ_ip(v4)_ CO_3_^2-^ | Carbonates [11] |
| 751 | 753 | 756 |  | β_op_ C-C_6ring_; ω CH | Indigo [16]; microbial alkyl halides, carboxylic acids and amines [22] |
| 723 | 725 |  | 723 | β_op_ *cis* CH;  ρ_ip_ CH_2_ crystalline;  β_ip_ C-C_6ring_; β_ip_ C-C_5ring_ | Indigo [16]; binders [17-19,23] |
| 697 | 698 | 698 | 702 | ω C=O; δ =CH  β_op_ C-C_6ring_;  β_op_ C-C_5ring_ | Indigo [16]; binders [17-19]; microbial alkyl halides, carboxylic acids and amines [22] |

where ν = stretching, δ = bending/scissoring, ρ = rocking, β = deformation modes, ω = wagging; breath = breathing vibrations; as and s = asymmetric and symmetric, respectively; ip and op = in plane and out of plane, respectively; iph and oph = in phase and out of phase, respectively; 6ring = a 6-membered ring of the indigo molecule; 5ring = a 5-membered ring of the indigo molecule; binders = IR vibrational modes shared between the diverse binders (i.e., proteinaceous binders, vegetable oils, waxes, and lacquers).

**Supplementary Table 2:** µATR-FTIR absorption bands of INV_13 and their attribution.

| **Wavenumber (cm^-1^)** | | | | | **Vibrational modes** | **Compounds** |
| --- | --- | --- | --- | --- | --- | --- |
| **INV_13_2** | **INV_13_3** | **INV_13_5** | **INV_13_8** | **INV_13_9** |  |  |
|  |  |  |  | 3423 | ν OH | Lignin [14] |
|  |  |  | 3403 |  | ν OH | *Urushi* lacquer [12-13] |
|  | 3378 |  |  |  | ν OH | Silicates [18] |
|  |  | 3350 |  |  | ν OH | Cellulose [14]; microbial polysaccharides and proteins [15] |
| 3324 |  |  |  |  | ν OH | Cellulose [26] |
|  |  |  | 3260 |  | ν NH | Microbial proteins [15]; Indigo [16]; proteinaceous binders [17] |
|  | 3233 | 3233 |  |  | ν OH | Carbonates [11] |
|  |  |  | 3068 |  | Amide II overtone | Proteinaceous binders [17] |
|  |  |  |  | 2970 | ν_as_ CH_3_ | Natural waxes [19] |
| 2924 | 2926 | 2923 | 2925 | 2920 | ν_as_ CH_2_ | Cellulose, lignin [14]; microbial lipids and polysaccharides [15]; binders [17-19] |
|  |  |  |  | 2879 | ν_s_ CH | Microbial lipids and polysaccharides [15]; binders [17-18] |
| 2850 | 2853 | 2853 | 2851 |  | ν_s_ CH_2_ | Microbial lipids and polysaccharides [15]; binders [17-19] |
|  | 1705 | 1703 | 1703 | 1710 | ν + δ SiO; ν C=O | Silicates [11], free fatty acids from waxes [20] |
| 1653 | 1653 | 1650 | 1650 | 1642 | ν C=O;  ν *cis* HC=CH;  δ OH (water) | Microbial α-helix proteins [15]; proteinaceous binders and oils [17]; lignin [21] |
|  | 1620 |  | 1626 |  | ν C=O;  β NH_2_ Amide II;  β OH (water) | *Urushi* lacquer [12-13]; Indigo [16]; proteinaceous binders [17]; microbial primary amines [22] |
|  |  |  | 1585 |  | ν_ip_ C-C_6ring_ | Indigo [16] |
| 1553 |  | 1546 |  | 1545 | δ NH; ν CN;  ν SiO | Silicates [11]; microbial α-helix proteins [15]; proteinaceous binders [17] |
|  | 1536 |  | 1536 | 1530 | δ NH; ν CN | Microbial α-helix proteins [15]; proteinaceous binders [17] |
|  |  |  | 1481 |  | ν C-C_6ring_;  ρ_s(iph)_ CH | Indigo [16] |
| 1463 | 1460 | 1458 | 1460 | 1454 | β scissoring CH_2_;  ν C-C_6ring_;  ρ_as(oph)_ CH; | Binders [12-13,18-19] lignin [14]; microbial lipids [15]; Indigo [16] |
|  | 1434 | 1437 | 1437 |  | ν_(v3)_ CO_3_^2-^;  δ_as_ (CH)CH_3;_  δ_s_ CH_2_ | Binders [17-19]; carbonates [23] |
| 1410 | 1405 | 1410 | 1417 | 1413 | ν CN; ρ NH | Indigo [16]; proteinaceous binders [17] |
| 1370 | 1377 | 1372 | 1375 | 1372 | δ_s_ CH_2_; δ_s_ CH | Cellulose, waxes [14]; vegetable oils [17] |
| 1318 | 1316 | 1320 | 1322 | 1326 | ω CH_2_  ν C-C_6ring_;  ν C-C_5ring_;  ρ_as(oph)_ CH | Indigo [16]; Cellulose, lignin [24] |
|  |  |  | 1296 |  | ρ_s(iph)_ CH;  β_ip_ C-C_5ring_ | Indigo [16]; binders [17-19] |
| 1261 | 1267 | 1260 | 1257 | 1268 | β guaiacyl ring;  β OH | Lignin [14] |
| 1204 |  | 1210 | 1201 | 1204 | ν P=O | Microbial phospholipids and nucleic acids [27] |
|  | 1190 | 1180 |  |  | ν C-C_5ring_;  ρ_as(oph)_ CH;  β_ip_ C-C_6ring_; ν CN | Indigo [16]; binders [17-19]; microbial aliphatic amines [22] |
|  |  | 1160 | 1168 |  | ν CN; ρ_as(oph)_ CH;  ρ NH; ν C-O;  ν SiO | Silicates [11]; Indigo [16]; triglyceride ester linkage in vegetable oils [17]; β_(1,3)_ glycosidic bonds [25] |
| 1152 |  |  |  | 1150 | ν_as_ C-O-C | Microbial glycogen and nucleic acids [15] |
| 1102 | 1102 | 1102 | 1105 | 1112 | ν_s(v1)_ CO_3_^2-^;  ν C-O; ν CN  ν_as(ip)_ glucose ring | Carbonates [11]; binders [17-19]; microbial aliphatic amines [22]; cellulose [24] |
| 1073 | 1082 | 1076 | 1073 | 1076 | ν_as_ SiO; ν C-O;  ν C-C_5ring_; ρ CO;  ν_s_ PO | Silicates [11]; binders [12-13,17]; microbial nucleic acids and phospholipids [15]; Indigo [16]; β_(1,3)_ glycosidic bonds [25] |
| 1053 |  |  |  |  | ν_as_ SiO; ν_as_ C-O  ν_s(v1)_ CO_3_^2-^; | Silicates and carbonates [11]; cellulose [14] |
| 1030 | 1032 | 1034 | 1040 |  | ν_s_ C-O | Cellulose [14,26]; binders [17-19]; orpiment [18]; β_(1,3)_ glycosidic bonds [25] |
| 1019 |  | 1010 |  |  | ν C-C_6ring_ breath._as_  ν_as_ SiO | Silicates [11]; Indigo [16] |
| 1003 |  | 1007 |  |  | ν C-C_6ring_ breath._as_  ν_as_ SiO | Silicates [11]; Indigo [16] |
|  |  |  | 975 | 980 | ν_as_ SiO;  β_op_ *trans* HC=CH | Silicates [11]; vegetable oils [17] |
| 930 |  |  |  | 935 | δ =CH | Microbial alkyl halides, carboxylic acids and amines [22]; β_(1,3)_ glycosidic bonds [25] |
|  | 909 | 915 | 911 |  | β-linkage glucose ring; β CH;  ν_as_ SiO | Silicates [11]; orpiment [18]; cellulose [26] |
| 880 |  |  | 880 |  | ρ_op(v2)_ CO_3_^2-^;  β CH;  β_ip_ C-C_6ring_;  β_ip_ C-C_5ring_ | Indigo [16]; vegetable oils and waxes [17-19]; carbonates [23] |
| 860 |  | 870 |  |  | ρ_op(v2)_ CO_3_^2-^ | Carbonates [23] |
|  |  |  |  | 840 | ρ_op(v2)_ CO_3_^2-^ | Carbonates [23] |
| 806 |  | 805 | 810 |  | Iα cellulose;  ρ_op(v2)_ CO_3_^2-^ | Carbonates [11]; orpiment [18]; cellulose [26] |
| 776 | 780 | 777 | 780 |  | ρ_ip(v4)_ CO_3_^2-^ | Carbonates [11] |
|  |  |  | 760 |  | β_op_ C-C_6ring_;  ω CH | Indigo [16]; microbial alkyl halides, carboxylic acids and amines [22] |
| 720 | 725 | 720 | 725 |  | β_op_ *cis* CH;  ρ_ip_ CH_2_ crystalline;  β_ip_ C-C_6ring_;  β_ip_ C-C_5ring_ | Indigo [16]; binders [17-19,23] |
| 700 | 700 | 698 | 700 | 703 | ω C=O; δ =CH  β_op_ C-C_6ring_;  β_op_ C-C_5ring_; | Indigo [16]; binders [17-19]; microbial alkyl halides, carboxylic acids and amines [22] |

where ν = stretching, δ = bending/scissoring, ρ = rocking, β = deformation modes, ω = wagging; breath = breathing vibrations; as and s = asymmetric and symmetric, respectively; ip and op = in plane and out of plane, respectively; iph and oph = in phase and out of phase, respectively; 6ring = a 6-membered ring of the indigo molecule; 5ring = a 5-membered ring of the indigo molecule; binders = IR vibrational modes shared between the diverse binders (i.e., proteinaceous binders, vegetable oils, waxes, and lacquers).

**Supplementary Table 3:** µATR-FTIR absorption bands of INV_15 and their attribution.

| **Wavenumber (cm^-1^)** | | **Vibrational modes** | **Compounds** |
| --- | --- | --- | --- |
| **INV_15_2** | **INV_15_4** |  |  |
|  | 3536 | ν OH | Carbonates [18] |
| 3518 |  | ν OH | Silicates [18] |
| 3393 | 3401 | ν OH | *Urushi* lacquer [12-13] |
| 3369 |  | ν OH | Silicates [18] |
| 3332 |  | ν OH | Cellulose [26] |
| 3320 |  | ν OH | Cellulose [26] |
| 3288 | 3290 | ν NH | Microbial proteins [15]; proteinaceous binders [17] |
|  | 3071 | Amide II overtone | Proteinaceous binders [17] |
| 2923 |  | ν_as_ CH_2_ | Cellulose, lignin [14]; microbial lipids and polysaccharides [15]; binders [17-19] |
|  | 2899 | ν_s_ CH | Microbial fatty acids [27] |
| 2851 |  | ν_s_ CH_2_ | Microbial lipids and polysaccharides [15]; binders [17-19] |
| 1650 | 1648 | ν C=O (also Amide I); ν *cis* HC=CH;  δ OH (water) | Microbial α-helix proteins [15]; proteinaceous binders and oils [17]; lignin [21] |
| 1621 | 1623 | ν C=O; β NH_2_ Amide II; β OH (water) | *Urushi* lacquer [12-13]; proteinaceous binders [17]; microbial primary amines [22] |
| 1543 | 1542 | δ NH; ν CN; ν SiO | Silicates [11]; microbial α-helix proteins [15]; proteinaceous binders [17] |
|  | 1513 | ν aromatic ring | Lignin [14] |
| 1450 |  | β scissoring CH_2_; ρ_as(oph)_ CH; | Binders [12-13,18-19] lignin [14]; microbial lipids [15] |
| 1428 | 1437 | ν_(v3)_ CO_3_^2-^; δ_as_ (CH)CH_3_; δ_s_ CH_2_ | binders [17-19]; carbonates [23] |
|  | 1408 | ν CN; ρ NH | Proteinaceous binders [17] |
| 1370 | 1365 | δ_s_ CH_2_; δ_s_ CH | Cellulose, waxes [14]; vegetable oils [17] |
| 1312 | 1315 | ω CH_2_: ρ_as(oph)_ CH | Cellulose, lignin [24] |
|  | 1279 | β guaiacyl ring; β OH | Lignin [14] |
| 1245 | 1248 | β syringyl ring | Lignin [14] |
| 1231 |  | ν C-O | Triglyceride ester linkage of vegetable oils [17] |
| 1198 |  | ρ_as(oph)_ CH; ν CN | Binders [17-19]; microbial aliphatic amines [22] |
| 1145 | 1142 | ν_as_ C-O-C | Microbial glycogen and nucleic acids [15] |
| 1114 | 1106 | ν_s(v1)_ CO_3_^2-^; ν C-O;  ν_as(ip)_ glucose ring; ν CN | Carbonates [11]; binders [17-19]; microbial aliphatic amines [22]; cellulose [24] |
|  | 1075 | ν_as_ SiO; ν C-O; ρ CO; ν_s_ PO | Silicates [11]; binders [12,17]; microbial nucleic acids and phospholipids [15]; β_(1,3)_ glycosidic bonds [25] |
| 1053 |  | ν_as_ SiO; ν_s(v1)_ CO_3_^2-^; ν_as_ C-O | Silicates and carbonates [11]; cellulose [14] |
| 1030 | 1027 | ν_s_ C-O; | Cellulose [14,26]; binders [17-19]; β_(1,3)_ glycosidic bonds [25] |
| 909 | 900 | β-linkage glucose ring; β CH; ν_as_ SiO; | Silicates [11]; cellulose [26] |
| 870 |  | ρ_op(v2)_ CO_3_^2-^ | Carbonates [23] |
| 799 | 793 | Iα cellulose; ρ_op(v2)_ CO_3_^2-^ | Carbonates [11]; cellulose [26] |
| 765 | 772 | ρ_ip(v4)_ CO_3_^2-^ | Carbonates [11] |
| 735 |  | β_op_ *cis* CH; ρ_ip_ CH_2_ crystalline; | Binders [17-19,23] |
| 712 | 713 | β_op_ *cis* CH; ρ_ip_ CH_2_ crystalline; | Binders [17-19,23] |
| 702 | 700 | ω C=O; δ =CH | Binders [17-19]; microbial alkyl halides, carboxylic acids and amines [22] |

where ν = stretching, δ = bending/scissoring, ρ = rocking, β = deformation modes, ω = wagging; breath = breathing vibrations; as and s = asymmetric and symmetric, respectively; ip and op = in plane and out of plane, respectively; iph and oph = in phase and out of phase, respectively; 6ring = a 6-membered ring of the indigo molecule; 5ring = a 5-membered ring of the indigo molecule; binders = IR vibrational modes shared between the diverse binders (i.e., proteinaceous binders, vegetable oils, waxes, and lacquers).

**Supplementary Table 4:** µATR-FTIR absorption bands of INV_20 and their attribution.

| **Wavenumber (cm^-1^)** | | **Vibrational modes** | **Compounds** |
| --- | --- | --- | --- |
| **INV_20_3** | **INV_20_4** |  |  |
| 3432 |  | ν OH | Lignin [14] |
|  | 3403 | ν OH | *Urushi* lacquer [12-13] |
| 2968 | 2968 | ν_as_ CH_3_ | Natural waxes [19] |
| 2927 | 2926 | ν_as_ CH_2_ | Cellulose, lignin [14]; microbial lipids and polysaccharides [15]; binders [17-19] |
| 2874 | 2877 | ν_s_ CH | Microbial lipids and polysaccharides [15]; binders [17-18] |
|  | 2850 | ν_s_ CH_2_ | Microbial lipids and polysaccharides [15]; binders [17-19] |
| 2804 |  | ν_s_ CH | Aliphatic compounds and binders [17-18] |
| 1719 | 1715 | ν + δ SiO; ν C=O | Silicates [11], free fatty acids from waxes [20] |
| 1648 | 1650 | ν C=O (also Amide I);  ν *cis* HC=CH; δ OH (water) | Microbial α-helix proteins [15]; proteinaceous binders and oils [17]; lignin [21] |
| 1455 | 1450 | β scissoring CH_2_; ν C-C_6ring_;  ρ_as(oph)_ CH; | Binders [12-13,18-19] lignin [14]; microbial lipids [15]; Indigo [16] |
| 1412 | 1405 | ν CN; ρ NH | Indigo [16]; proteinaceous binders [17] |
| 1370 | 1371 | δ_s_ CH_2_; δ_s_ CH | Cellulose, waxes [14]; vegetable oils [17] |
| 1328 | 1328 | ω CH_2_; ν C-C_6ring_;  ν C-C_5ring_; ρ_as(oph)_ CH | Indigo [16]; Cellulose, lignin [24] |
| 1270 | 1261 | β guaiacyl ring; β OH | Lignin [14] |
| 1201 |  | ν P=O; ν_as(3)_ SO_4_; β Fe-OH | Microbial phospholipids and nucleic acids [27]; green vitriol [28-30] |
| 1141 | 1150 | ν_as_ C-O-C | Microbial glycogen and nucleic acids [15] |
| 1114 | 1120 | ν_as(3)_ SO_4_; β Fe-OH | Green vitriol [28-30] |
| 1074 | 1077 | ν_as_ SiO; ν C-O; ρ CO;  ν C-C_5ring_; ν_s_ PO | Silicates [11]; binders [12-13,17]; microbial nucleic acids and phospholipids [15]; Indigo [16]; β_(1,3)_ glycosidic bonds [25]; green vitriol [28-29] |
| 1050 | 1054 | ν_as_ SiO; ν_s(v1)_ CO_3_^2-^;  ν_as_ C-O; ν_as(3)_ SO_4_; β Fe-OH | Silicates and carbonates [11]; cellulose [14]; green vitriol [28-29] |
| 980 | 980 | ν_as_ SiO; ν_s(1)_ SO_4_;  β_op_ *trans* HC=CH | Silicates [11]; vegetable oils [17], green vitriol [28-29] |
| 940 | 930 | δ =CH; ν_as(1)_ AsOFe | Microbial alkyl halides, carboxylic acids and amines [22]; β_(1,3)_ glycosidic bonds [25]; degradation product of orpiment (iron arsenate) [31] |
|  | 867 | ρ_op(v2)_ CO_3_^2-^ | Carbonates [23] |
|  | 853 | ν AsO | Degradation product of orpiment (calcium arsenate) [32] |
| 843 | 847 | ρ_op(v2)_ CO_3_^2-^; ν_as(2)_ AsOFe | Carbonates [23]; degradation product of orpiment (iron arsenate) [31] |
| 823 | 830 | ρ_op(v2)_ CO_3_^2-^; β CH;  β_ip_ C-C_5,6rings_; ν FeO; ν_s(1)_ AsOFe | Indigo [16]; vegetable oils [17]; carbonates [23]; α-glycosidic linkage [25]; degradation product of orpiment (iron arsenate) [31]; green vitriol [33] |
| 750 |  | β_op_ C-C_6ring_; ω CH | Indigo [16]; microbial alkyl halides, carboxylic acids and amines [22] |
| 720 | 710 | β_op_ *cis* CH; β_ip_ C-C_5ring_  ρ_ip_ CH_2_ crystalline; β_ip_ C-C_6ring_ | Indigo [16]; binders [17-19,23] |

where ν = stretching, δ = bending/scissoring, ρ = rocking, β = deformation modes, ω = wagging; breath = breathing vibrations; as and s = asymmetric and symmetric, respectively; ip and op = in plane and out of plane, respectively; iph and oph = in phase and out of phase, respectively; 6ring = a 6-membered ring of the indigo molecule; 5ring = a 5-membered ring of the indigo molecule; binders = IR vibrational modes shared between the diverse binders (i.e., proteinaceous binders, vegetable oils, waxes, and lacquers).


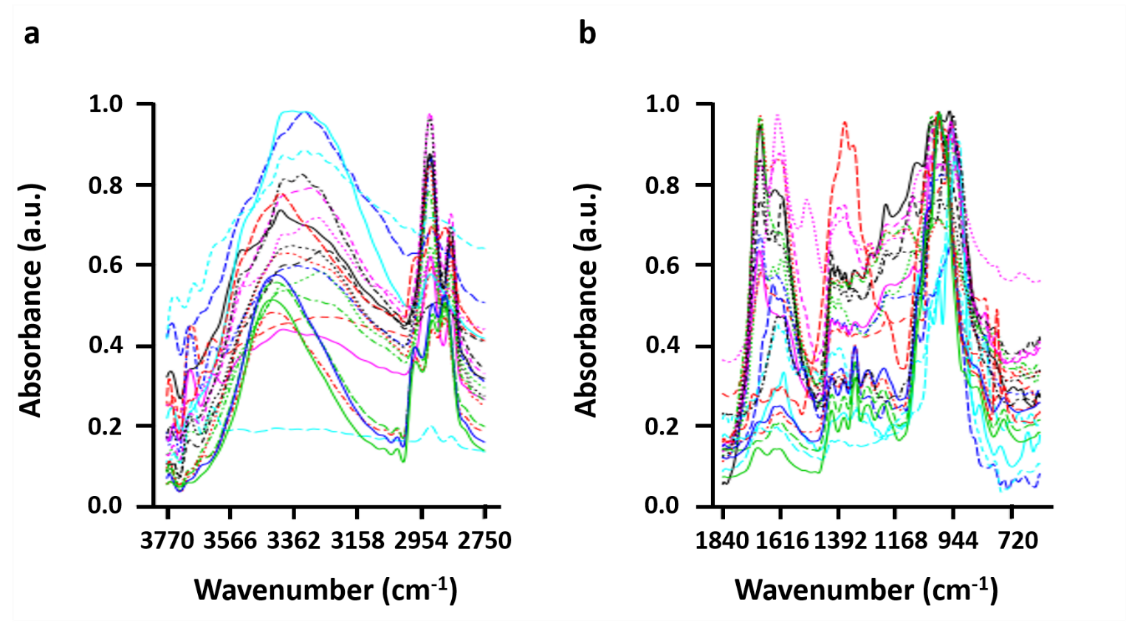


**Supplementary Fig 1:** Smoothed µATR-FTIR spectra divided in the two intervals **a)** 3770-2750 cm^-1^ and **b)** 1840-720 cm^-1^, on which the statistical analysis was performed.


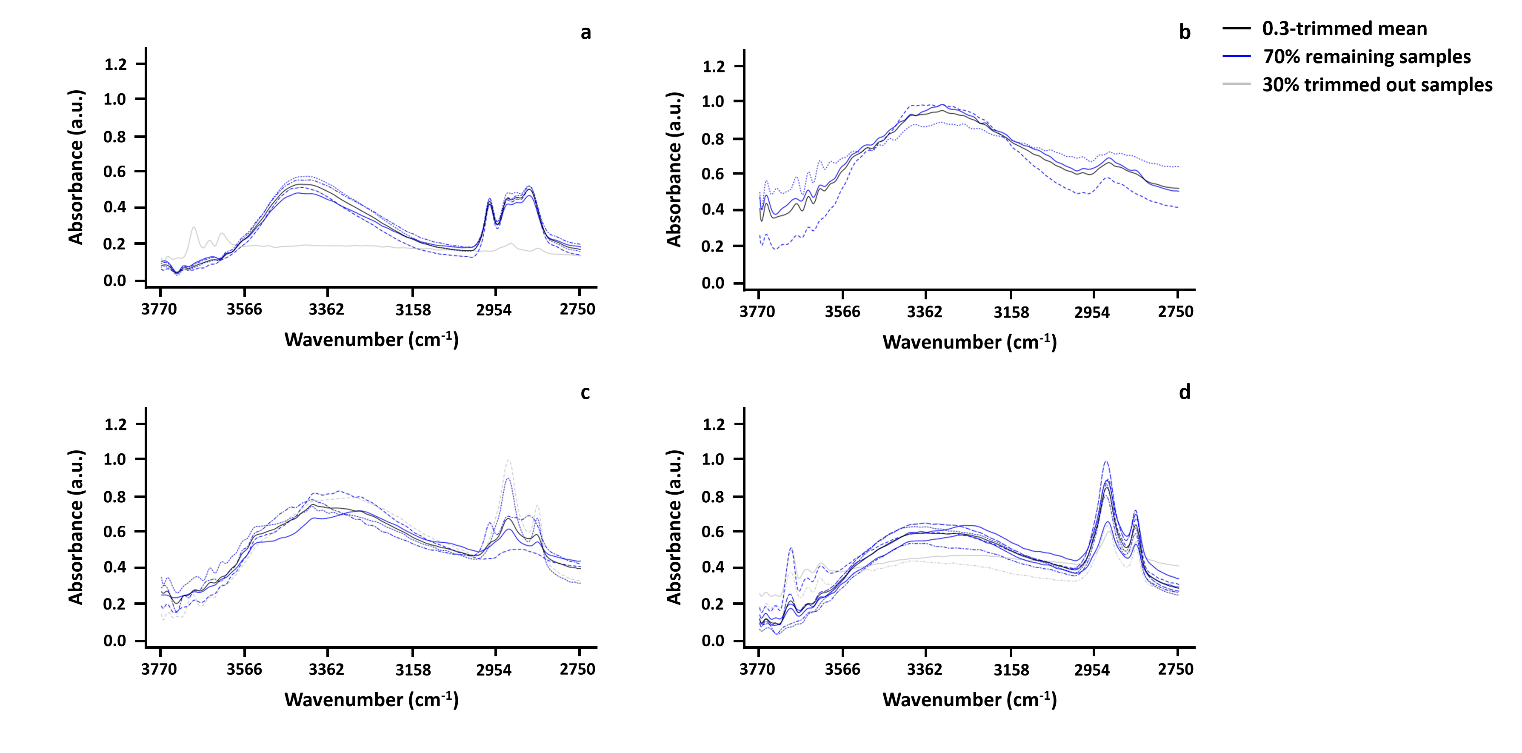
 **Supplementary Fig 2:** Arrangement of µATR-FTIR spectra in the 3770-2750 cm^-1^ interval belonging to **a)** first, **b)** second, **c)** third, and **d)** four clusters based on the Modified Band Depth (MBD) function, which identified the deepest curve (i.e., 0.3-trimmed mean), the core (70% remaining samples) and the most external spectra (30% trimmed out samples) of the clusters.


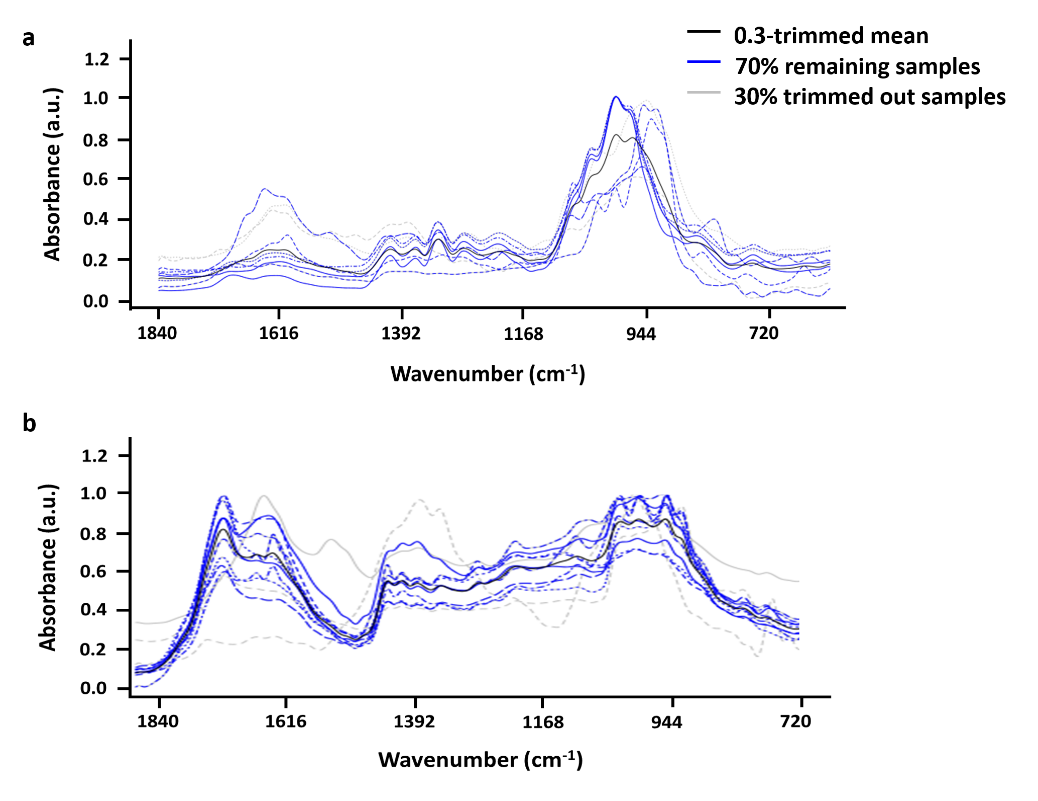


**Supplementary Fig 3:** Arrangement of µATR-FTIR spectra in the 1840-719 cm^-1^ interval belonging to **a)** first, and **b)** second clusters based on the Modified Band Depth (MBD) function, which identified the deepest curve (i.e., 0.3-trimmed mean), the core (70% remaining samples) and the most external spectra (30% trimmed out samples) of the clusters.

**
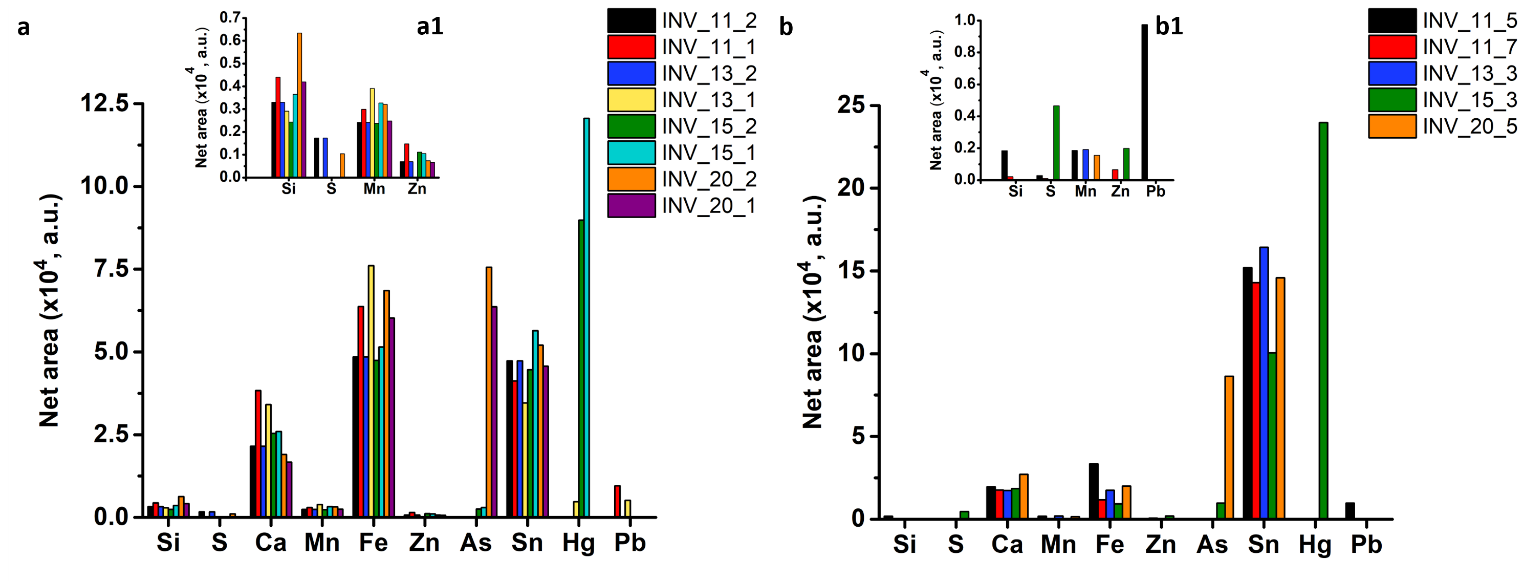
**

**Supplementary Fig 4:** Elemental composition of the **a)** *verso* and **b)** background *recto* of the four studied wallpapers obtained by performing X-ray Fluorescence (XRF) spectroscopy. In **a)**, the labels _1 and _2 indicate the written and non-written portions of the *verso*, respectively; for clarity, the inlets **a1)** and **b1)** showed the distribution of the trace elements detected for each sampling point.


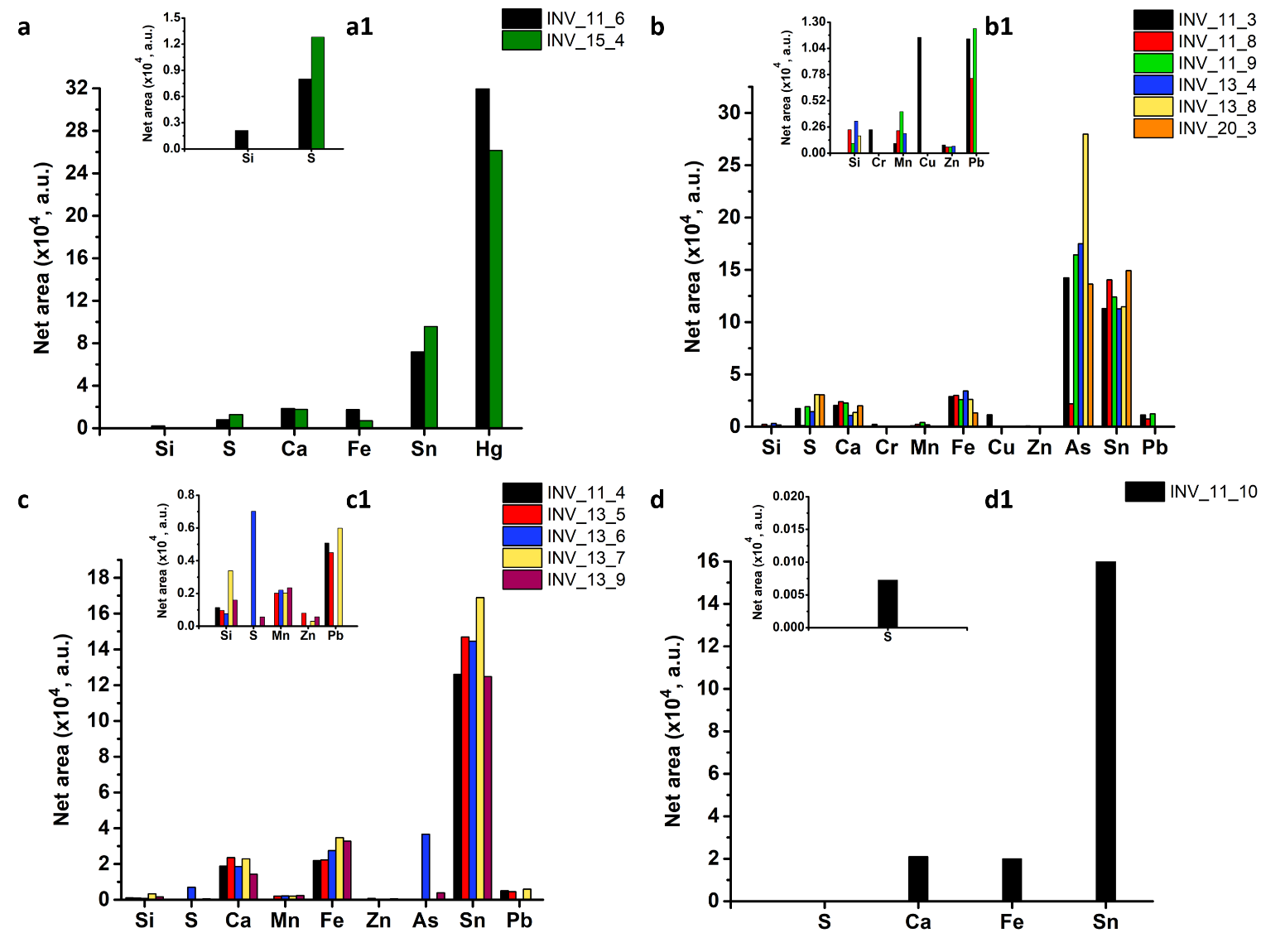


**Supplementary Fig 5:** Elemental composition of the **a)** red, **b)** green, **c)** brown, and **d)** black painted portions of the wallpapers’ *recto* obtained by performing X-ray Fluorescence (XRF) spectroscopy. For clarity, the inlets **a1)**, **b1)**, **c1)**, and **d1)** showed the distribution of the trace elements detected for each sampling point.

**References**

[1] Wood, S. N. Stable and efficient multiple smoothing parameter estimation for generalized additive models. *J. Am. Stat. Assoc.* **99**, 673-686 (2004).

[2] Srivastava, A., Klassen, E. P. Functional and shape data analysis (eds. Srivastava, A., Klassen, E. P.) (Springer-Verlag New York, 2016).

[3] Ramsay, J. O., Wicham, H., Graves, S., Hooker, G. fda: Functional Data Analysis. R package version 2.4.8.1. https://cran.r-project.org/web/packages/fda (2020).

[4] Maechler, M., Rousseeuw, P., Struyf, A., Hubert, M., Hornik, K. cluster: Cluster Analysis Basics and Extensions. R package version 2.1.0. https://cran.r-project.org/web/packages/cluster (2019).

[5] Galili, T. dendextend: an R package for visualizing, adjusting, and comparing trees of hierarchical clustering. *Bioinformatics*. **31**, 3718-3720 (2015).

[6] Lopez-Pintado, S., Torrente, A. depthTools: Depth Tools Package. R package version 0.4. https://cran.r-project.org/web/packages/depthTools (2013).

[7] Coy, M. R., *et al*. Nested quantitative PCR approach with improved sensitivity for the detection of low titer levels of *Candidatus Liberibacter asiaticus* in the Asian citrus psyllid, *Diaphorina citri Kuwayama*. *J. Microbiol. Methods.* **102**, 15-22 (2014).

[8] Op De Beeck, *et al*. Comparison and validation of some ITS primer pairs useful for fungal metabarcoding studies, *PLOS one*. **9**, e97629 (2014).

[9] Yoon, S. H., *et al*. Introducing ExBio-Could: a taxonomically united database of 16s rRNA and whole genome assemblies. *Int. J. Syst. Evol. Microbiol.* **67**, 1613-1617 (2016).

[10] Altschul, S. F., *et al*. Gapped BLAST and PSI-BLAST: a new generation of protein database search programs. *Nucleic Acids Res.* **25**, 3389-3402 (1997).

[11] Miliani, C., Rosi, F., Daveri, A., Brunetti, B. G. Reflection infrared spectroscopy for the non-invasive in situ study of artists’ pigments. *Appl. Phys. A.* **106**, 295-307 (2012).

[12] Niimura, N., Miyakoshi, T. Structural study of oriental lacquer films during the hardening process. *Talanta* **70**, 146-152 (2006).

[13] Niimura, N., Miyakoshi, T. Characterization of natural resin films and identification of ancient coating. *J. Mass. Spectrom. Soc. Jap.* **51** 439-457 (2003).

[14] Poli, T., Chiantore, O., Nervo, M., Piccirillo, A. Mid-IR fiber-optic reflectance spectroscopy for identifying the finish on wooden furniture. *Anal. Bioanal. Chem.* **400**, 1161-1171 (2011).

[15] Naumann, D., Helm, D., Labischinski, H. Microbial characterizations by FT-IR Spectroscopy. *Nature*. **351**, 81-82 (1991).

[16] Baran, A., Fiedler, A., Schulz, H., Baranska, M. *In situ* Raman and IR spectroscopic analysis of indigo dye. *Anal. Methods.* **2**, 1372-1376 (2010).

[17] Meilunas, R. J., Bentsen, J. G., Steiberg, A. Analysis of aged paint binders dye FTIR spectroscopy. *Stud. Conserv.* **35**, 33-51 (1990).

[18] Vahur, S., *et al.* ATR-FT_IR spectral collection of conservation materials in the extended region of 4000-80 cm^-1^. *Anal. Bioanal. Chem.* **408**, 3373-3379 (2016).

[19] Tanner, N., Lichtenberg-Kraag, B. Identification and quantification of single and multi-adulteration of beeswax by FTIR-ATR spectroscopy. *Eur. J. Lipid Sci. Technol.* **121**, 1900245 (2019).

[20] Svecnjak, L., *et al*. An approach for routine analytical detection of beewax adulteration using FTIR-ATR spectroscopy. *J. Apic. Sci.* **59**, 37-49 (2015).

[21] Sidi-Yacoub, B., Oudghiri, F., Belkadi, M., Rodriguez-Barroso, R. Characterization of lignocellulosic components in exhausted sugar beet pulp waste by TG/FTIR analysis. *J. Therm. Anal. Calorim.* **138**, 1801-1809 (2019).

[22] Kanjana, M., Kanimozhi, G., Panneerselvam, A. Fourier Transform Infrared spectroscopy (FTIR) analysis of some isolated endophytic fungi. *G.J.B.B.* **8**, 73-79 (2019).

[23] Moretti, P., *et al*. Materials and techniques of twentieth century Argentinean murals. *Proc. Chem.* **8**, 221-230 (2013).

[24] Liu, Y., Kim, H. J. Fourier Transform Infrared Spectroscopy (FT-IR) and simple algorithm analysis for rapid and non-destructive assessment of developmental cotton fibers. *Sensors (Basel).* **17**, 1469 (2017).

[25] Tsai, C. H., Yen, Y. H., Yang, J. P. W. Finding of polysaccharide-peptide complexes in *Cordyceps militaris* and evaluation of its acetylcholinesterase inhibition activity. *J. Food Drug Anal.* **23**, 63-70 (2015).

[26] Abidi, N., Cabrales, L., Hequet, E. Fourier transform infrared spectroscopic approach to the study of the secondary cell wall development in cotton fiber. *Cellulose*. **17**, 309-320 (2010).

[27] Gupta, B. S., Jelle, B. P., Gao, T. Application of ATR-FTIR spectroscopy to compare the cell materials of wood decay fungi with wood mould fungi. *Int. J. Spectrosc.* **2015**, 521938 (2015).

[28] Rouchon, V., *et al*. Raman and FTIR spectroscopy applied to the conservation report of paleontiological collections: identification of Raman and FTIR signatures of several iron sulfate species such as ferrinatrite and sideronatrite. *J. Raman Spectrosc.* **43**, 1265-1274 (2012).

[29] Corregidor, V., Viegas, R., Ferreira, L. M., Alves, L. C. Study of Iron gall inks, ingredients and paper composition using non-destructive techniques. *Heritage*. **2**, 2691-2703 (2019).

[30] Ursescu, M., Malutan, T., Ciovica, S. Iron gall inks influence on papers’ thermal degradation FTIR spectroscopy applications. *Eur. J. Sci. Theol.* **3**, 71-84 (2009).

[31] Brechbuhl, Y., Christl, I., Elzinga, E. J., Kretzschmar, R. Competitive sorption of carbonate and arsenic to hematite: Combined ATR-FTIR and batch experiments. *J. Colloid Interf. Sci.* **377**, 313-321 (2012).

[32] Vermeulen, M., *et al*. Visualization of As(III) and As(IV) distributions in degraded paint micro-samples from Baroque- and Rococo-era paintings. *J. Anal. At. Spectrom.* **31**, 1913-1921 (2016).

[33] Rajalakshmi, P., *et al*. Analytical studies on *Annabethi Chenthuram*, a Siddha herbomineral formulation. *Ind. J. Pharm. Sci.* **79**, 987-993 (2017).
